# A diastereoselective Scholl reaction: point-to-helical chirality transfer in molecular nanographenes†

**DOI:** 10.1039/d5sc02563j

**Published:** 2025-06-09

**Authors:** Sergio Marcos López, Manuel Buendia, Israel Fernández, Salvatore Filippone, Nazario Martín

**Affiliations:** a Departamento de Química Orgánica I, Facultad de Ciencias Químicas, Universidad Complutense Avd. Complutense, S/N Madrid 28040 Spain israel@quim.ucm.es salvatorefilippone@ucm.es nazmar@ucm.es; b IMDEA-Nanociencia C/ Faraday, 9, Campus de Cantoblanco Madrid 28049 Spain

## Abstract

Stereoselective control of molecular nanographene helicity has been achieved by a point-to-helical chirality transfer during the Scholl graphitization reaction to obtain compound 4. Density functional theory calculations indicate that the complete diastereoselective process takes place mainly under kinetic control. Circular dichroism and circularly polarised luminescence studies of both enantiomers show the expected mirror images, with a dissymmetry factor (*g*_lum_) value of 1.0 × 10^−3^ determined at 470 nm and a circularly polarized luminescence brightness (*B*_CPL_) of 4.9 M^−1^ cm^−1^.

## Abstract 2

A stereogenic center directs the bending of molecular nanographene during the graphitization process, enabling stereoselective control of helicity *via* point-to-helical chirality transfer in the Scholl reaction.

## Graphical abstract

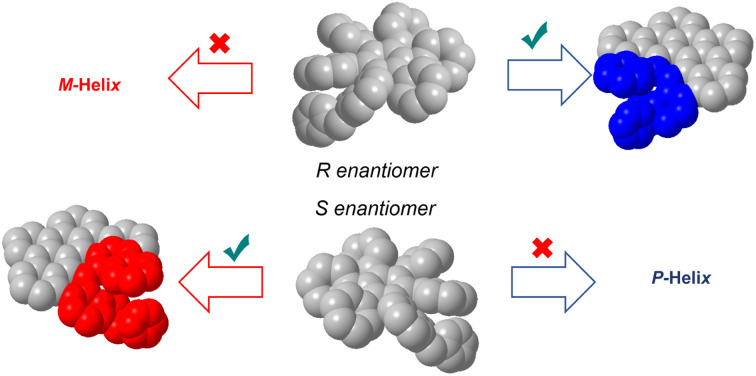

## Introduction

Carbon nanostructures endowed with chiral elements have not been equally developed, depending primarily on the nanoform of carbon. Thus, whereas chirality has been extensively studied in 0D fullerenes, this is not the case for 1D carbon nanotubes and more recently 2D graphene.^1^ In particular, chirality in graphene is mostly related to the presence of defects in the planar surface. However, despite the rapid development of the chemistry of pristine graphene^2^ and its derivatives, namely graphene oxide (GO) and reduced graphene oxide (r-GO), the application of stereoselective synthetic methods has only been poorly addressed using relatively small graphene fragments (molecular nanographenes), thus lacking a further dimension of control.^3^

Molecular nanographenes are a fascinating class of polycyclic aromatic hydrocarbons, usually ranging in size from 1 to 5 nm, bridging the gap between small, simple fused arene systems and larger nanographene sheets (up to 100 nm).^4^ According to their size and shape, a variety of synthetic methods have been employed in their bottom-up fabrication, allowing the development of a wide variety of diverse and extraordinary carbon-based molecules with well-defined structures able to extend beyond planarity.^5^ Among these, nanographene molecules endowed with inherent structural defects producing chirality have garnered significant attention.^6^ Indeed, their asymmetrically bent π-conjugated structures are a source of unique chiroptoelectronic properties such as chiral-induced spin selectivity (CISS)^7^ and circularly polarized luminescence (CPL).^8–11^

The synthesis of molecular nanographenes requires a multistep protocol that usually leads to polyarylbenzene derivatives as synthetic precursors. These, in turn, undergo a further graphitization process through the Scholl dehydrogenation reaction, involving multiple oxidative ring closures.^12,13^ Performing the Scholl reaction on appropriately functionalized polyarylbenzenes can also result in curved structures.^14,15^

For example, *ortho* substituents on peripheral arenes can inhibit planarization, promoting out-of-plane graphitization, leading to chiral structures with helicoidal stereogenic elements. Controlling the stereochemistry of these elements not only prevents the formation of stereoisomeric mixtures but also enables the tuning of their electronic and optical properties. Thus, stereoselective synthetic methodologies that precisely guide the formation and control of stereogenic elements, affording inherently chiral nanographenes, are currently highly demanded.^16^

**Fig. 1:**
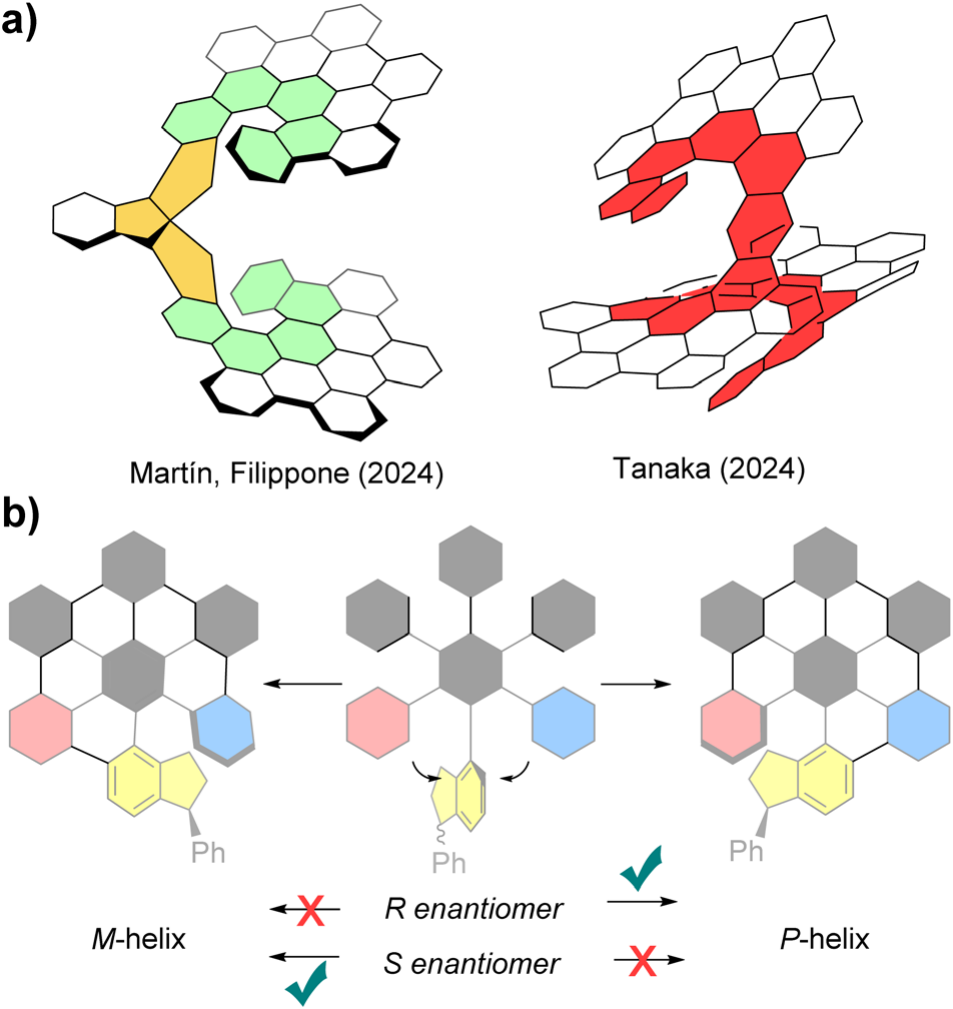
(a) First examples of enantioselective synthesis of molecular nanographenes; (b) the presence of point chirality in a polyarylbenzene precursor (*R* or *S* enantiomer) could afford two different diastereomers from the formation of *M*- or *P*-helix in the Scholl graphitization reaction.

Recently, we successfully achieved the pioneering enantioselective synthesis of a chiral nanographene in both enantiomeric forms, without relying on chiral HPLC separation of racemates or the use of chiral starting materials (Fig. 1a, left).^10^ The adopted strategy was based on the asymmetric introduction of a stereogenic centre and, thereafter, on the transfer of this point chirality to a stereogenic axis (spiro compound) and, eventually, to helicity of the nanographene moiety, which represented the first example of an enantiospecific Scholl reaction. To the best of our knowledge, only one other recent example of enantioselective synthesis of nanographenes has been reported (Fig. 1a, right).^17^

We subsequently investigated the scope of this synthetic strategy and its effectiveness for less rigid systems lacking the chiral spiro central core of triindane. For this purpose, our strategy focused on the presence of only one single chiral center in the polyarylbenzene precursor. The challenge of directing the nanographene bending and transferring the chiral information in the graphitization step has not been thoroughly addressed so far (Fig. 1b). Thus, herein we report on the transfer of point chirality to helicoidal chirality in the Sholl reaction and, in particular, the ability of a single stereogenic center to successfully direct the asymmetric folding of the nanographene structure.

## Results and discussion

**Scheme 1:**
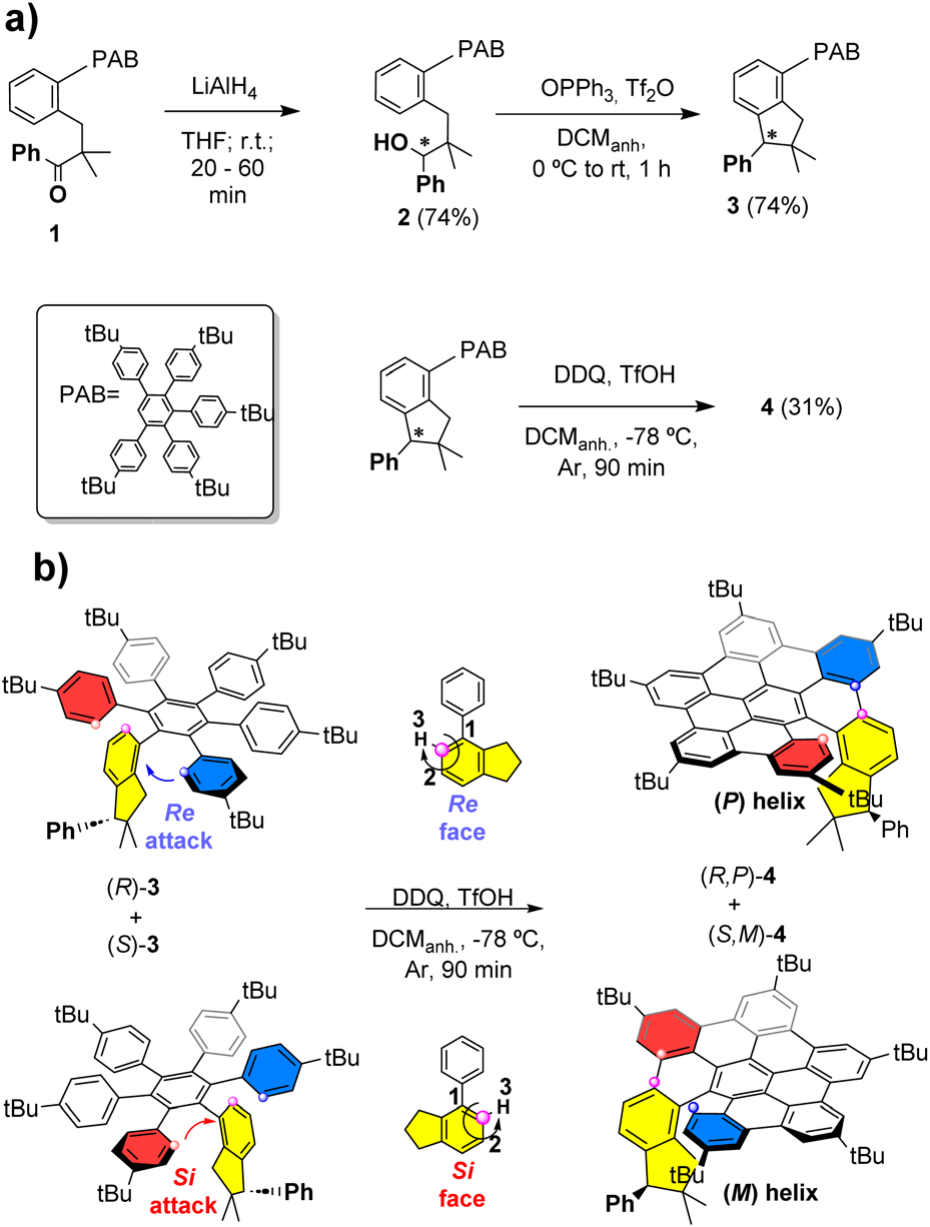
(a) General scheme for the synthesis of nanographene 4; (b) detail of the point-to-helical chirality transfer in the stereoselective Scholl graphitization for each enantiomer of racemate 3.

We initially designed hexarylbenzene 3 to be synthesized in two steps from ketone 1, in which one of the six arenes features an *ortho* substitution pattern, in such a way that avoids the planarization of the final nanographene (see the ESI†) (Scheme 1a).

Ketone 1 was, in turn, obtained in a three-step synthetic process from commercially available isobutyrophenone and 2-iodobenzyl bromide (see the ESI†). Thus, the ketone reduction was carried out using LiAlH_4_, affording the racemic secondary alcohol 2 in 74% yield (Scheme 1a). In the next step, an indane unit, endowed with a stereogenic center, is generated as the pivotal one of the six aromatic rings in the polyarylbenzene systems. This occurs through an intramolecular Friedel–Crafts reaction of benzylic alcohol 2 promoted by triphenylphosphine oxide and triflic anhydride (Hendrickson reagent) with a 74% yield.^18^

Here, in sharp contrast with our previously reported rigid triindane structure,^10^ the absence of other stereogenic elements does not help to fully preserve the chiral information. Thus, we decided to explore the effect of the stereogenic centre on the graphitisation process using the racemic mixture.

The ability of this new chiral center to direct the helical bending of the final nanographenes was demonstrated during the Scholl reaction. Indeed, even by generating another stereogenic element, a single diastereoisomer was recovered for 4 with a 31% yield, as a pair of (*R*,*P*) and (*S*,*M*) enantiomers (see Scheme 1b). Formation of diastereomers (*R*,*M*)-4 and (*S*,*P*)-4 was totally excluded based on the careful analyses of reaction products both in crude form and after chromatographic purification on silica gel. Furthermore, HPLC studies under different conditions, including different chiral columns and detectors capable of measuring circular dichroism, consistently confirmed the presence of only two peaks showing chiroptical activity corresponding to both (*R*,*P*)-4 and (*S*,*M*)-4 enantiomers. The relative configuration of 4 was assigned by ^1^H ROESY-NMR experiments, where, along with other through–space correlations, a spatial proximity between the protons of the indane phenyl group and those of the near *tert*-butyl group is observed (see the ESI†).

It can then be proposed that, in the racemic mixture, the arrangement of aryl groups in enantiomer (*R*)-3 allows the nucleophilic (blue-labelled aryl) attack from the Re face of the indane, while shielding the Si face. As a result, (*R*,*P*)-4 is formed with complete stereospecificity. Conversely, for the (*S*)-3 enantiomer, the oxidative coupling takes place from the Si face (red-labelled aryl), affording exclusively (*S*,*M*)-4 (Scheme 1b).

**Fig. 2:**
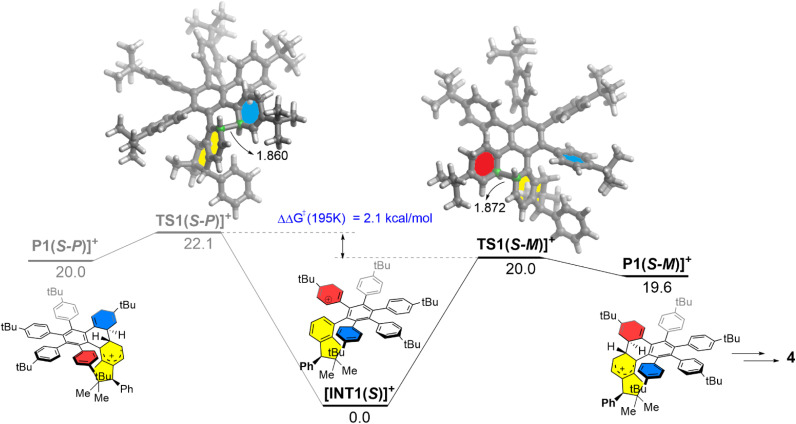
Computed reaction profiles for the key Scholl cyclization step occurring at the beginning of the graphitization process. Relative free energies (Δ*G*, at 195 K) and bond distances are given in kcal mol^−1^ and angstroms, respectively. All data were computed at the CPCM-RI-B3LYP-D3(BJ)/def2-SVP level.

To further support the above rationalization, density functional theory (DFT) calculations were performed at the dispersion-corrected CPCM-RI-B3LYP-D3(BJ)/def2-SVP level (see the ESI† for computational details). The computed reaction profile for the key Scholl cyclization step involving the (*S*)-3 precursor is shown in Fig. 2, which gathers the corresponding relative free energies (Δ*G*) computed at 195 K (*i.e.*, the temperature at which the reaction was carried out). We investigated the two possible cyclization pathways leading to the corresponding (*S*,*P*) and (*S*,*M*) diastereomers and starting from the protonated intermediate [INT1(*S*)]^+^, therefore, following the so-called arenium cation mechanism.^19^

Interestingly, we found that the formation of the (*S*,*M*) product (black profile) is both kinetically and thermodynamically favored over the formation of its (*S*,*P*) diastereoisomer (grey profile). Moreover, the computed free energy barrier difference, ΔΔ*G*^≠^ (195 K) = 2.1 kcal mol^−1^, is translated into a >99 : 1 diastereomeric ratio, according to the Boltzmann distribution at 195 K, which is fully consistent with the exclusive formation of the (*S*,*M*)-4 nanographene observed experimentally (see the ESI† for details)

**Fig. 3:**
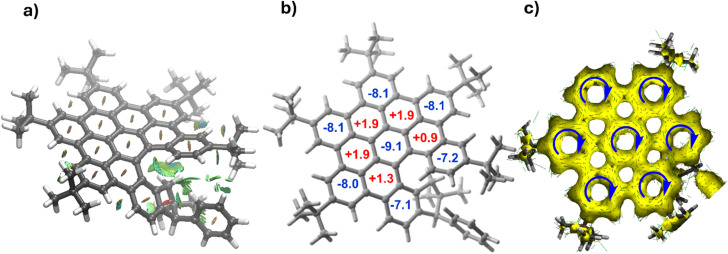
(a) Contour plots of the reduced density gradient isosurfaces (density cutoff of 0.045 a.u.) for (*S*,*M*)-4. The green surfaces indicate attractive noncovalent interactions. (b) Computed NICS values (in ppm). (c) AICD plot (isosurface value of 0.04 a.u.).

From a topological point of view, the newly prepared helical nanographenes are directly related to hexa-*peri*-hexabenzocoronene (HBC). Despite this, we were curious to investigate the influence of the indenyl fragment on the structure and properties of these species. First, the indenyl moiety not only forces the observed helicity but also stabilizes the system by means of noncovalent interactions. Indeed, the NCIPlot^20^ method applied to (*S*,*M*)-4 confirms the occurrence of stabilizing CH⋯π interactions mainly involving the CH_2_ moiety of the indenyl fragment and adjacent aryl rings (see Fig. 3a). Moreover, we assessed the aromaticity of the system by computing the Nuclear Independent Chemical Shift (NICS)^21^ values of the different six-membered rings. As shown in Fig. 3b, NICS calculations indicate strong aromaticity for both the central and peripheral six-membered rings, whereas the inner rings connecting them can be considered as non-aromatic (or weakly antiaromatic). This is confirmed by the Anisotropy of the Induced Current Density (AICD) method,^22^ which clearly shows the occurrence of diatropic (*i.e.*, aromatic) induced currents within the central and peripheral six-membered rings (Fig. 3c). This situation strongly resembles that found in the parent HBC,^23^ thus confirming the strong relationship between HBC and the helical nanographenes prepared herein.

**Fig. 4:**
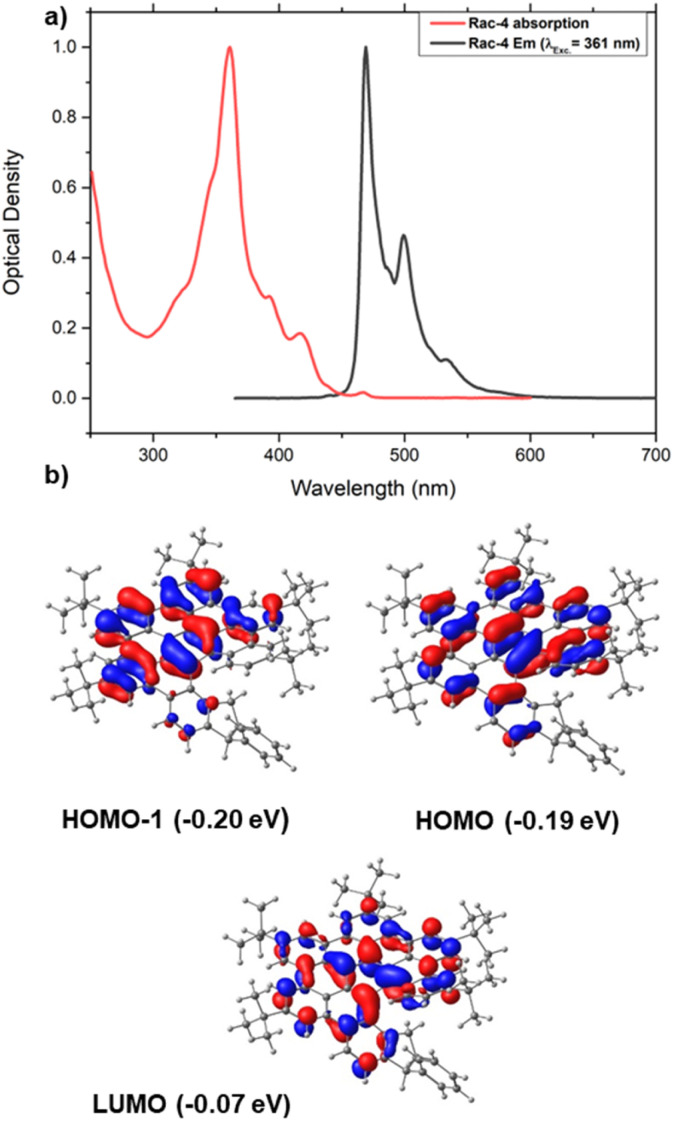
(a) UV/vis absorption spectra of racemic 4 and normalized fluorescence emission spectra (black line) in CH_2_Cl_2_ (1.8 μM) upon irradiation at the absorption maximum. (b) Computed molecular orbitals of (*S*,*M*)-4 involved in the lower energy absorptions (isosurface value of 0.03 a.u.).

Optical properties of compound 4 were studied by UV-vis and fluorescence spectroscopy and were found to be coherent with other previously reported related nanographenes (Fig. 4).^10,24^ Thus, 4 features an absorption band with a maximum at 361 nm along with two weaker bands at *ca.* 400 nm (392 and 418 nm), which further resembles the situation found in other polyaromatic systems, such as hexa-*tert*-butyl hexabenzocoronene (HBC). Indeed, our time-dependent (TD) DFT calculations on (*S*,*M*)-4 also assign the lower energy absorptions to the HOMO-1 → LUMO and HOMO → LUMO vertical transitions, respectively, which as shown in Fig. 4, involve π-molecular orbitals fully delocalized within the HBC moiety, therefore confirming the π–π* nature of the absorptions. Moreover, analogously to HBC-like molecules having a helicene moiety, the emission spectrum of 4 displays two bands at 469 and 500 nm and a shoulder at 532 nm.^24^

HPLC separation of racemic 4 resulted in the levorotatory (−)-4, as the first eluted enantiomer (see the ESI†), and the dextrorotatory (+)-4 eluted thereafter. For helicenes, a general correspondence between helicity and specific rotation is well established, according to which negative optical rotation is associated with a left-handed absolute configuration *M*, while dextrorotatory helicenes exhibit a right-handed helicity (*P*).^25–27^ Therefore, considering that 4 has a [5]helicene substructure, we can safely assign the configuration (*S*,*M*) to (−)-4, while a (*R*,*P*) absolute configuration is assigned to (+)-4.

**Fig. 5:**
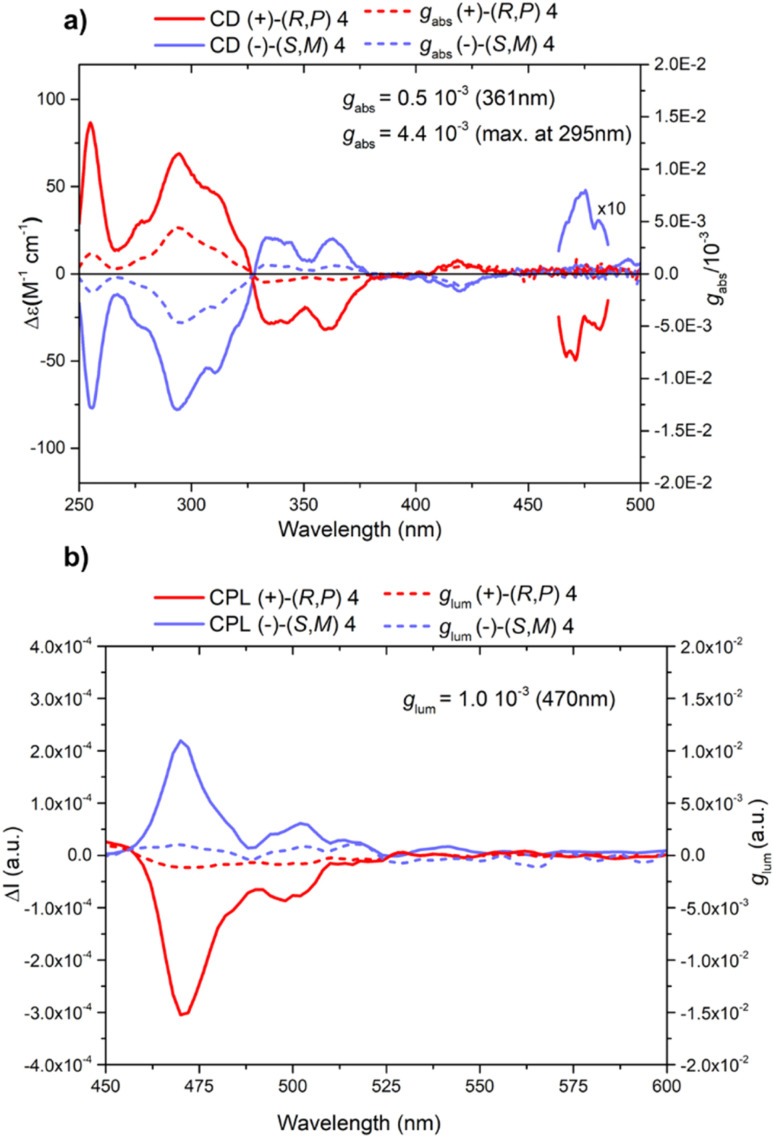
(a) Circular dichroism absorption spectra (solid lines) for both enantiomers (+)-(*R*,*P*)-4 (in red) and (+)-(*S*,*M*)-4 (in blue) (CHCl_3_, 20 °C, 6 μM) and absorptive dissymmetry factor (*g*_abs_) (dashed lines); (b) CPL spectra (solid lines) of (+)-4 (in red) and (−)-4 (in blue) and luminescence dissymmetry factor (*g*_lum_) (dashed lines) (*λ*_exc_ = 390 nm, CHCl_3_, 20 °C, ∼1 μM).

This result has also been confirmed through the study of circular dichroism (CD) and circularly polarised luminescence (CPL) spectra. CD and CPL spectra of both enantiomers show the expected mirror image. The CD spectra show two main bisignate signals at around 327 nm and 390 nm, with two strong peaks at 255 and 293 nm, two bands of medium intensity centered at 340 and 360 nm, and two small peaks at 420 and 469 nm (Fig. 5a). As expected, these spectra are very similar to the CD spectra of the analogous two-layer nanographene previously reported by us, holding (−)-4 the same sign as the enantiomer with helicity *M*, whose absolute configuration was assigned by X-ray diffraction.^10^ In addition, the same positive Cotton effect between 298 nm and 340 nm, and the negative peak at around 300 nm found in the CD spectrum of (*M*)-[5]helicene support the (*S*,*M*) assignment for the absolute configuration of (−)-4.^28^ Moreover, the CPL spectrum presents the same sign as the very small peaks at 469 nm of the CD spectra, which is positive for (−)-4 (Fig. 5a and b, blue lines) with peaks at 470 and 500 nm. The luminescence dissymmetry factor *g*_lum_ value at 470 nm is 1.0 × 10^−3^ while the circularly polarized luminescence brightness (*B*_CPL_) is 4.9 M^−1^ cm^−1^.

## Conclusions

In summary, we have carried out the synthesis of a new molecular nanographene (4) through enantioselective control of the helicity generated by a point-to-helical chirality transfer during the last synthetic step involving a Scholl graphitization reaction. Interestingly, only a single diastereomer is produced in the transformation as a pair of (*R*,*P*) and (*S*,*M*) enantiomers. The complete diastereoselective process takes place, according to our DFT calculations, mainly under kinetic control. Indeed, the free energy barrier difference, ΔΔ*G*^≠^ (195 K) = 2.1 kcal mol^−1^, computed for the key cyclization step, is consistent with the observed >99 : 1 diastereomeric ratio.

Notably, the topology and aromaticity of the newly prepared helical nanographenes strongly resemble those of the parent HBC and related helical systems. Moreover, the *g*_lum_ value determined at 470 nm was 1.0 × 10^−3^, which also compares well to that of other related molecular nanographenes, resulting in a circularly polarized luminescence brightness value of *B*_CPL_ = 4.9 M^−1^ cm^−1^.

Work is currently in progress in order to improve the control over the enantioselectivity in these amazing systems.

## Author contributions

S. M. L. and M. B. designed and performed the experiments; I. F. carried out the computational study. S. F. and N. M. conceived the idea and co-wrote the first draft. All authors have participated in the editing and review process of the manuscript.

## Conflicts of interest

There are no conflicts to declare.

## Supplementary Material

SC-016-D5SC02563J-s001

## Data Availability

The data supporting this article have been included as part of the ESI.†
