# Supplementary material for: A diastereoselective Scholl reaction: point-to-helical chirality transfer in molecular nanographenes
Source: Chem Sci. 2025 Jun 9;16(28):12867–72. doi: 10.1039/d5sc02563j (PMC12183460; doi:10.1039/d5sc02563j)
Supplement: SC-016-D5SC02563J-s001 [file SC-016-D5SC02563J-s001.pdf]

Supplementary Information (SI) for Chemical Science.
This journal is © The Royal Society of Chemistry 2025

Supporting Information

Diastereoselective Scholl reaction: Point-to-helical chirality transfer in molecular nanographenes

Sergio Marcos López,[a] Manuel Buendia,[a] Israel Fernández,*[a] Salvatore Filippone,*[a] and Nazario Martín*[a,b]

Table of Contents

General 2
General procedure 3
NMR spectra 7
^{1}H ROESY Experiment and Diastereomeric Assignment 17
HPLC separation 21
Chiral Dichroism and CPL espectra 22
Photophysical Studies 23
Computational Details 25
Stereoselectivity in a reaction under kinetic control 42

General

Unless otherwise noted, all materials including solvents were obtained from commercial suppliers and used without further purification. 2,3,4,5-tetrakis(4-(*tert*-butyl)phenyl)cyclopenta-2,4-dien-1-one was prepared according to the procedure reported in the literature.[1] All reactions were performed with dry solvents (dried by filtration through alumina according to the method described)[2] and under an atmosphere of argon in dried glassware with standard vacuum-line techniques. Microwave reactions were performed in an Anton-Parr Monowave 300 microwave reactor. Mechanochemical reactions were performed in a Retsch GmbH MM 200 miller.

All work-up and purification procedures were carried out with reagent-grade solvents in air. Silica column chromatography was conducted with Scharlau 40-60 µm silica gel. Analytical thin-layer chromatography (TLC) was performed using E. Merck silica gel 60 F254 precoated plates (0.25 mm).

Analytical HPLC monitoring was performed on an Agilent Technologies 1260 Infinity HPLC system, using (*R*,*R*) ULMO (5µm, 100Å, 25 cm x 4.6 mm ID) as analytical columns. The matrix used for MALDI-TOF was trans-2-[3-(4-tert-butylphenyl)-2-methyl-2-propenylidene]-malononitrile (DCTB) and mass analysis were performed in a Bruker Ultraflex II using a LTB MNL 106 laser source.

^{1}H NMR spectra were recorded at 300 MHz (Bruker AVIII), and ^{13}C NMR spectra were recorded at 75 (Bruker AVIII). Chemical shifts for ^{1}H NMR and ^{13}C NMR are expressed in parts per million (ppm) relative to the solvent. Deuterated chloroform ($CDCl_3$) was used as NMR solvent for all the compounds, the residual solvent signal ($CDCl_3$ δ 7.26 ppm for ^{1}H and δ 77.16 ppm for ^{13}C) was used for referencing of NMR spectra Data are reported as follows: chemical shift, multiplicity (s = singlet, d = doublet, dd = doublet of doublets, t = triplet, q = quartet, m = multiplet,), coupling constant (Hz), and integration.

UV-vis data was obtained on a Shimadzu UV-3600 spectrometer. Optical rotations were measured using an Anton Paar MCP 100 Polarimeter. Fluorescence spectra and fluorescence quantum yield measurements were obtained in a FluoTime 300 fluorescence spectrometer, using 1 cm pathlength quartz cells at 298 K.

The ECD spectra were measured on a JASCO J-1500 CD Spectrometer, over a spectral range of 250 nm to 500 nm in chloroform (ca. 10^{-6} M solutions). Measurements were made in a quartz cell with a 1 cm path length using a scanning speed of 50 nm/min, a response time of 4 seconds and standard instrument sensitivity. The CPL spectra were measured on a JASCO CPL-300 with a 180º geometry. The following parameters were used: excitation and emission slit width of 1 nm, integration time of 4 seconds, scan speed of 50 nm/min, with 10 accumulations. (ca. 10^{-6} M solutions).

[1] D. Lungerich, J. F. Hitzenberger, M. Marcia, F. Hampel, T. Drewello and N. Jux, N. *Angew. Chem. Int. Ed.,* 2014, **53**, 12231.

[2] A. B. Pangborn, M. A. Giardello, R. H. Grubbs, R. K. Rosen and F. J. Timmers, *Organometallics*, 1996, **15**, 1518.

General procedure

3-(2-iodophenyl)-2,2-dimethyl-1-phenylpropan-1-one (5)

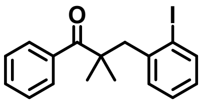

Isobutyrophenone (8,97 mmol, 1.33 g), 2-iodobenzyl bromide (13.46 mmol, 4 g) and potassium *tert*-butoxide (13.46 mmol, 1.51 g) were placed in a 25 mL zirconium oxide ball mill reactor along with twenty-two zirconium oxide 4 mm-diameter grinding balls. The reaction was carried out in a miller at 25 Hz for 6 hours. Upon completion, the reaction mixture was purified by a silica gel chromatography column using Hexane:DCM (2:1) as eluent. After removal of the solvents under reduced pressure **5** was obtained as a colorless oil (3.02 g, 91%).

^{1}H NMR (300 MHz, $CDCl_3$): δ (ppm): 7.85 (dd, *J*= 7.9, 1.2 Hz, 1H), 7.68-7.57 (m, 2H), 7.49-7.36 (m, 3H), 7.22 (td *J*= 7.7, 1.2 Hz, 1H), 7.14 (dd *J*= 7.7, 1.8 Hz, 1H), 6.88 (ddd, *J*= 7.9, 7.1, 1.8 Hz, 1H), 3.36 (s, 3H), 1.37 (s, 6H).

^{13}C{^{1}H} NMR (75 MHz, $CDCl_3$): δ (ppm): 209.2, 141.3, 139.9, 139.0, 130.7, 130.6, 128.1, 128.0, 127.9, 127.6, 103.2, 49.6, 48.4, 26.0.

HRMS ESI: Calculated for $C_{17}H_{17}IO$: $[M]^+$=364.0324 m/z; found: $[M+H]^+$=365.0407 m/z.

3-(2-((4-(tert-butyl)phenyl)ethynyl)phenyl)-2,2-dimethyl-1-phenylpropan-1-one (6)

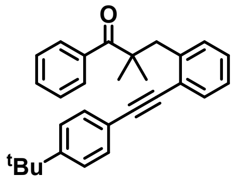

5 (1.37 mmol, 500 mg), tetrakis(triphenylphosphine) palladium(0) (0.068 mmol, 79.3 mg), and copper iodide (0.068 mmol, 13.0 mg) were added into a 30 mL microwave reaction vessel with a magnetic bar and dissolved in previously distilled Et_3N (8 mL). This solution was purged with an argon flow for 20 minutes, and 4-*tert*-butylphenylacetylene (2.06 mmol, 325.8 mg, 0.37 mL) was added just before the vessel was introduced into the microwave reactor, stirring the mixture for 3 h at 120°C and 600 rpm. The resulting mixture was diluted with DCM and was washed with a NH_4Cl saturated solution. The organic phase was dried with $MgSO_4$, the solvent was removed under reduced pressure, and the crude mixture was purified by silica gel chromatography column using Hexane:DCM (2:1) as eluent. **6** was obtained as an orange oil (473 mg, 87%).

^{1}H NMR (300 MHz, $CDCl_3$): δ (ppm): 7.67-7.61 (m, 2H), 7.56-7.52 (m, 1H), 7.48-7.36 (m, 2H), 7.41-7.36 (m, 3H), 7.33-7.27 (m, 2H), 7.24-7.15 (m, 3H), 3.41 (s, 2H), 1.36 (s, 6H), 1.35 (s, 9H).

$^{13}C\{^{1}H\}$ **NMR** (75 MHz, $CDCl_3$): δ (ppm): 209.6, 151.6, 139.9, 139.2, 132.3, 131.1, 130.8, 130.6, 128.0, 127.8, 127.6, 126.4, 125.4, 124.6, 120.3, 93.0, 88.7, 49.6, 42.9, 34.8, 31.1, 25.5

HRMS (ESI): Calculated for $C_{29}H_{30}O$: $[M]^+$=394.2297 m/z, found $[M+H]^+$=395.2375 m/z

3-(4''-(tert-butyl)-3',4',5',6'-tetrakis(4-(tert-butyl)phenyl)-[1,1':2',1''-terphenyl]-2-yl)-2,2-dimethyl-1-phenylpropan-1-one (1)

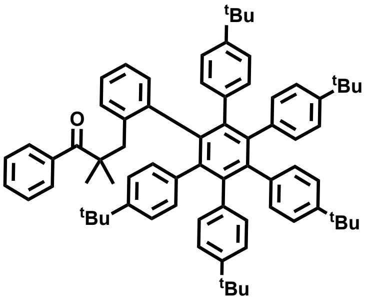

In a 30 mL microwave reaction vessel, **6** (0.532 mmol, 210.0 mg) and 2,3,4,5-tetrakis(4-(*tert*-butyl)phenyl)cyclopenta-2,4-dien-1-one (0.798 mmol, 486.1 mg) were placed along with a magnetic stir bar. Both solids where stirred for 40 min at 280°C and 600 rpm in a microwave reactor, the crude mixture was purified by silica gel chromatography column using Hexane:DCM (3:1) as eluent. After the removal of the solvent under reduced pressure, **1** was obtained as a brown solid (260 mg, 50%).

^{1}**H NMR** (300 MHz, $CDCl_3$): δ (ppm): 7.57-7.50 (m, 2H), 7.49-7.42 (m, 1H), 7.41-7.33 (m,2H), 7.01-6.97 (m, 1H), 6.87-6.55 (m, 23H), 3.07 (s, 2H), 1.12-1.07 (3s, 45H), 1.01 (s, 6H).

$^{13}C\{^{1}H\}$ **NMR** (75 MHz, $CDCl_3$): δ (ppm): 211.9, 147.8, 147.5, 147.4, 141.2, 140.8, 140.7, 140.2, 139.7, 138.9, 138.2, 138.1, 137.7, 135.8, 133.6, 131.4, 131.1, 130.6, 130.4, 128.1, 127.63, 127.6, 126.7, 125.9, 124.2, 123.3, 123.1, 123.1, 47.6, 43.0, 34.1, 31.3, 31.2, 26.1.

HRMS (MALDI-TOF): Calculated for $C_{73}H_{82}O$: $[M]^+$=974.6366 m/z, found: $[M]^+$=974.6352 m/z.

3-(4''-(tert-butyl)-3',4',5',6'-tetrakis(4-(tert-butyl)phenyl)-[1,1':2',1''-terphenyl]-2-yl)-2,2-dimethyl-1-phenylpropan-1-ol (2)

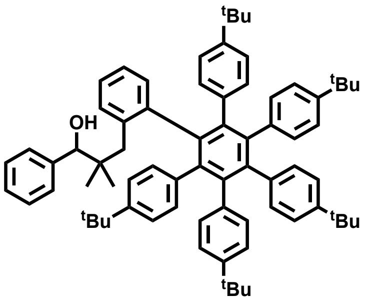

In a 25 mL two-neck round bottom flask, provided with a magnetic bar and connected to a condenser on one neck, $LiAlH_4$ (0.062 mmol, 2.33 mg) was suspended on anhydrous THF (5 mL) under an argon atmosphere. To this suspension, a solution of **1** (0.041 mmol, 40.0 mg) in anhydrous THF (2 mL) was added dropwise for 5 minutes and heated to reflux for 1 h. The

reaction was quenched with a HCl 10% aqueous solution (10 mL), the organic phase was washed with water and brine, dried with $MgSO_4$ and the solvent was removed under reduced pressure. The reaction crude was purified by silica chromatography column using Hexane:DCM (4:1) as eluent. After removal of the solvents under reduced pressure, **2** was obtained as a white solid (30 mg, 74%).

^{1}H NMR (300 MHz, $CDCl_3$): δ (ppm): 7.38-7.28 (m, 5H), 7.05 (dd, *J*= 7.4, 1.8 Hz, 1H), 7.00 (dd, *J*= 7.4, 1.6 Hz, 1H), 6.89-6.51 (m, 22H), 4.53 (s, 1H), AB system (δ_A=2.46, δ_B=2.30, *J*= 15.2 Hz, 2H), 1.80 (s, 1H), 1.12-1.07 (4s, 45H), 0.87 (s, 3H), 0.82 (s, 3H).

^{13}C{^{1}H} NMR (75 MHz, $CDCl_3$): δ (ppm): 147.5, 147.5, 147.3, 147.2, 141.8, 141.3, 140.7, 140.7, 140.5, 140.2, 140.0, 138.9, 138.1, 138.1, 138.0, 137.5, 137.4, 137.4, 133.6, 131.4, 131.0, 130.8, 128.9, 127.9, 127.4, 127.2, 125.3, 123.6, 122.9, 122.7, 82.1, 41.0, 39.4, 34.0, 31.1, 31.1, 23.5, 23.5.

HRMS (MALDI-TOF): Calculated for $C_{73}H_{84}O$: $[M]^+$=976.6522 m/z, found: $[M]^+$=976.6484 m/z

4,7,10,13,16-penta-tert-butyl-20,20-dimethyl-21-phenyl-20,21-dihydro-19H-dibenzo[fg,ij]benzo[9,10]pyreno[5,4,3,2,1-pqrst]cyclopenta[a]pentaphene (3)

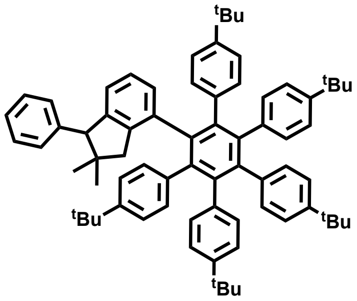

To a 5 mL round bottom flask, under argon atmosphere and provided with a magnetic stir bar, freshly distilled Tf_2O (0.29 mmol, 83 mg, 50 µL) was added to a solution of $OPPh_3$ (0.74 mmol, 206 mg) in anhydrous DCM (1 mL) at 0°C generating a white precipitate which was stirred for 30 min at room temperature. The white solid was added to a solution of **2** (0.031 mmol, 30 mg) in anhydrous DCM (2 mL) at 0°C in another 5 mL round bottom flask. After 15 min the reaction was quenched with MeOH, the organic phase was washed with brine, dried with $MgSO_4$ and the solvent was removed under reduced pressure. The final product was purified by preparative TLC using Hexane:CS_2 (1:4) as eluent, affording **3** as a white solid (22 mg, 74%).

^{1}H NMR (300 MHz, $CDCl_3$): δ (ppm): 7.16 (d, *J*= 7.2 Hz, 3H), 6.92-6.57 (m, 25H), 3.65 (s, 1H), AB system (δ_A = 2.57, δ_B =2.42, *J*= 15.5Hz, 2H), 1.25-1.05 (3s, 45H), 0.73 (s, 3H), 0.36 (s, 3H).

^{13}C{^{1}H} NMR (75 MHz, $CDCl_3$): δ (ppm): 147.70, 147.57, 147.35, 147.29, 144.96, 142.51, 142.08, 140.44, 140.30, 139.92, 138.87, 138.08, 138.02, 137.60, 131.11, 130.97, 130.52, 129.12, 128.23, 127.98, 127.82, 127.60, 125.84, 125.02, 122.94, 62.12, 46.28, 43.92, 34.08, 34.04, 34.02, 31.20, 29.55, 25.00.

HRMS (MALDI-TOF): Calculated for $C_{73}H_{82}$: $[M]^+$=958.6417 m/z, found $[M]^+$= 958.6376 m/z

4,7,10,13,16-penta-tert-butyl-20,20-dimethyl-21-phenyl-20,21-dihydro-19H-dibenzo[fg,ij]benzo[9,10]pyreno[5,4,3,2,1-pqrst]cyclopenta[a]pentaphene (4)

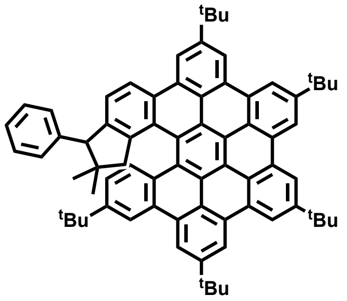

To a 50 mL round bottom flask, under argon atmosphere and provided with a magnetic stir bar, **3** (0.004 mmol, 4 mg), DDQ (0.024 mmol, 5 mg), and anhydrous DCM (4 mL) were added, the reaction mixture was cooled to -78°C and stirred for 5 minutes. Then, under argon bubbling, TfOH was added dropwise (20 µL) and the reaction was carried out keeping a constant temperature of -78 ºC and the argon bubbling for 25 minutes. After that time, the reaction was quenched with a $NaHCO_3$ saturated solution, the crude was extracted with DCM and washed with a $NaHCO_3$ saturated solution and brine. The organic phase was dried with $MgSO_4$, and the solvent was removed under reduced pressure. The crude was purified by silica gel chromatography column using Hexane:DCM (6:1) as eluent, affording **4** as a yellow solid (1.2 mg, 31%).

^{1}H NMR (300 MHz, $CDCl_3$): δ (ppm): 9.26 (d, *J*= 1.44 Hz, 1H), 9.25-9.21 (m, 3H), 9.15 (s, 1H), 9.11 (s, 1H), 9.07 (s, 1H), 9.03 (s, 1H), 8.75 (d, *J*= 2.0 Hz, 1H), 8.70 (d, *J*=8.3 Hz, 1H), 8.36 (d, *J*=8.5Hz, 1H), 7.49 (d, *J*= 8.2Hz, 1H), 7.47 (dd, *J*= 8.6, 2.0 Hz, 1H), 7.39 (dd, *J*= 7.1 Hz, 2H), 7.29 (dd, *J*= 7.4 Hz, 1H) 7.18 (d, *J*= 7.4 Hz, 1H), 3.78 (s, 1H), AB system (δ_A = 2.16, δ_B = 1.77, *J*= 16.3Hz, 2H), 1.82-1.75 (m, 36H), 1.47 (s, 9H), 0.54 (s, 3H), 0.42 (s, 3H).

^{13}C{^{1}H} NMR (176 MHz, $CDCl_3$) δ 149.47, 149.01, 148.90, 145.99, 142.99, 141.95, 131.18, 130.57, 130.52, 130.38, 130.12, 130.07, 129.73, 129.43, 128.30, 128.03, 126.03, 125.48, 124.29, 124.06, 124.01, 123.65, 123.45, 123.42, 123.08, 122.88, 121.55, 120.84, 120.54, 119.93, 119.43, 119.06, 118.59, 118.52, 77.18, 77.00, 76.82, 62.84, 49.17, 42.88, 35.71, 32.01, 31.92, 31.47, 31.23, 30.59, 29.70, 29.37, 25.68, 22.69, 14.13, 1.01.

HRMS (MALDI-TOF): Calculated for $C_{73}H_{72}$: $[M]^+$= 948.5634 m/z; found: $[M]^+$=948.5593 m/z.

The two enantiomers of product **4** have been isolated by HPLC separation with (*R,R*) ULMO (5 µm, 100 Å, 25 cm x 4.6 mm ID), Hex:DCM (95:5), 1 mL/min, 25°C, λ = 360 nm (see below Figure S9). The first eluted compound presents a $[\alpha]^{20}_D$ = −1212.3° (c=1.65 x 10^{-4}, $CHCl_3$; corresponding to **(*S,M*)-4**) and the second eluted enantiomers $[\alpha]^{20}_D$ = 1164.1° (c=2,58 x 10^{-4}, $CHCl_3$; corresponding to **(*R,P*)-4**)

NMR spectra

5. ^{1}H, 300MHz, $CDCl_3$, 298 K.

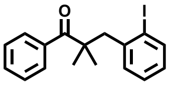

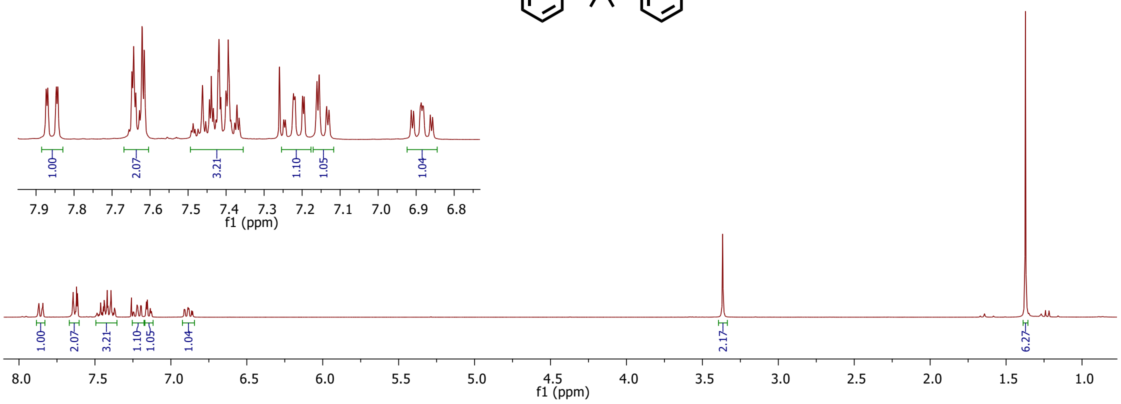

5. ^{13}C {^{1}H},75MHz, $CDCl_3$, 298 K.

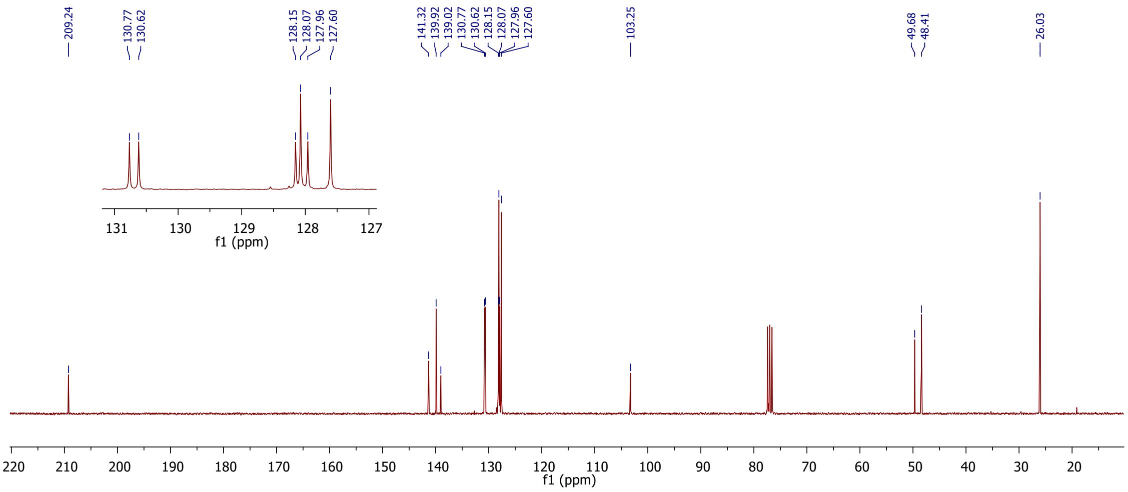

5. DEPT, 75MHz, $CDCl_3$, 298 K.

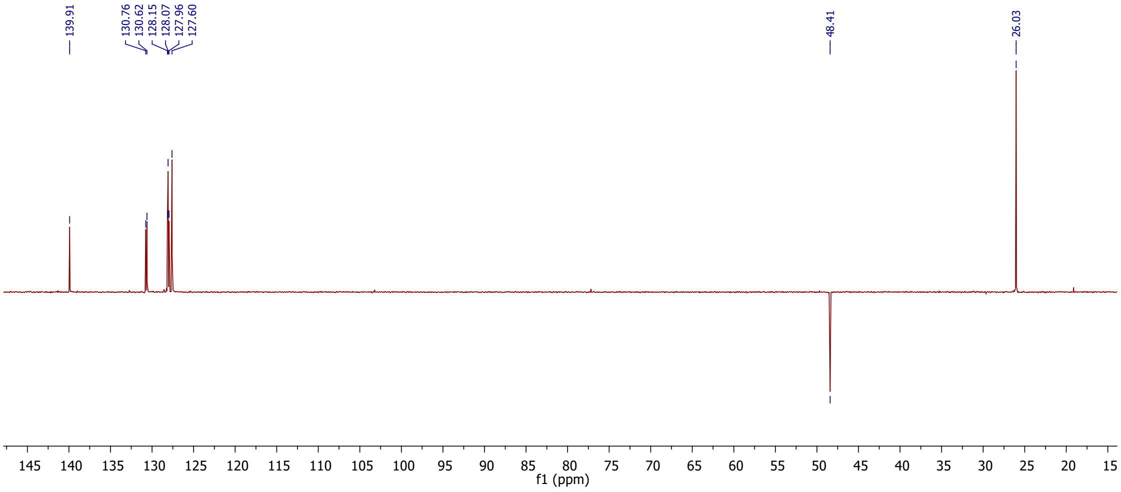

6. ^{1}H, 300MHz, $CDCl_3$, 298 K.

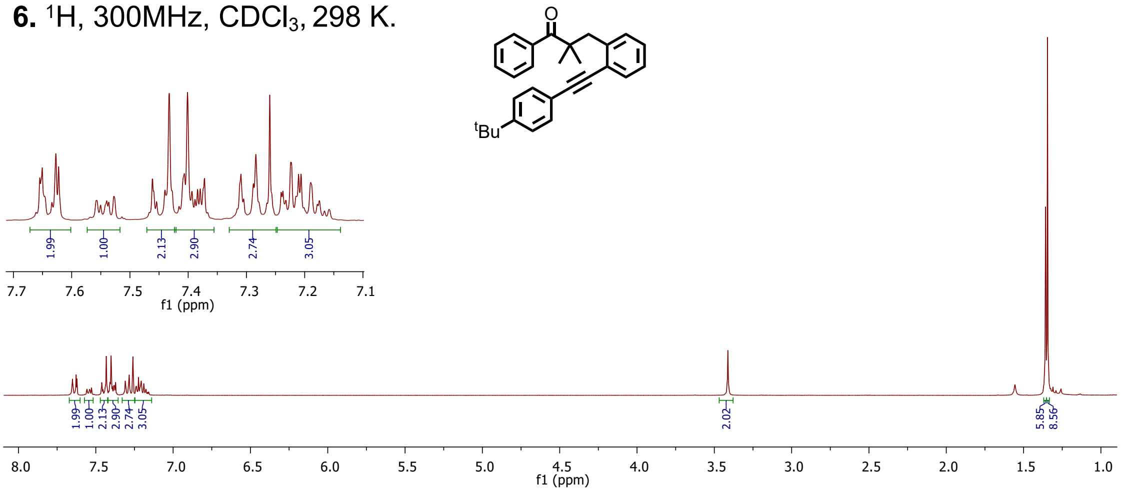

6. ^{13}C {^{1}H},75MHz, $CDCl_3$, 298 K.

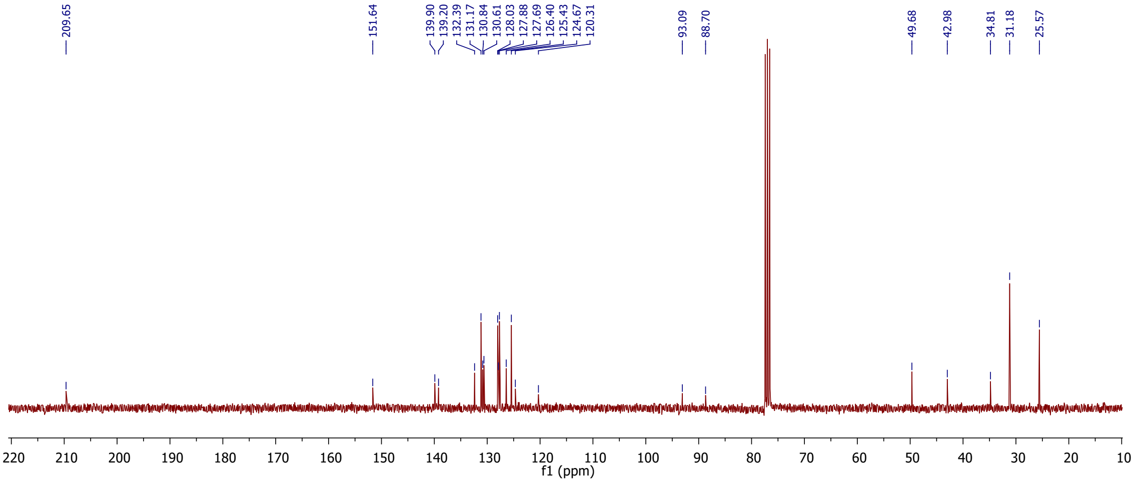

6. DEPT, 75MHz, $CDCl_3$, 298 K.

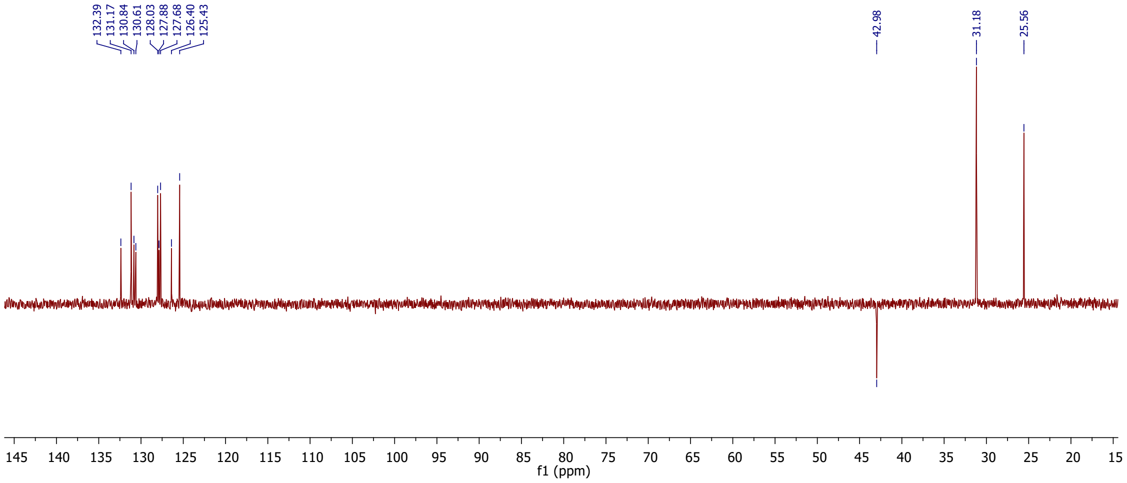

1. ^{1}H, 300MHz, $CDCl_3$, 298 K.

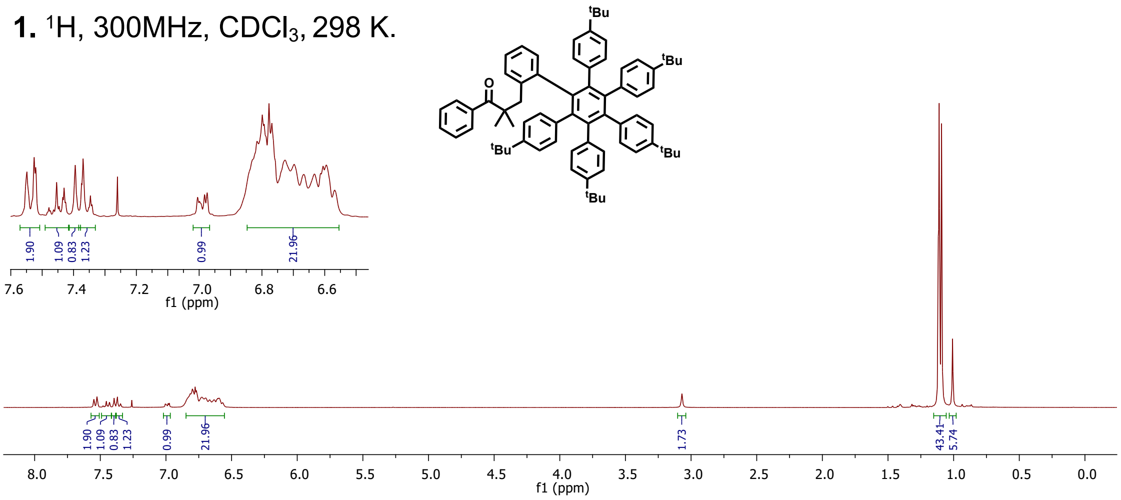

1. $^{13}C\ \{^1H\}$,75MHz, $CDCl_3$, 298 K.

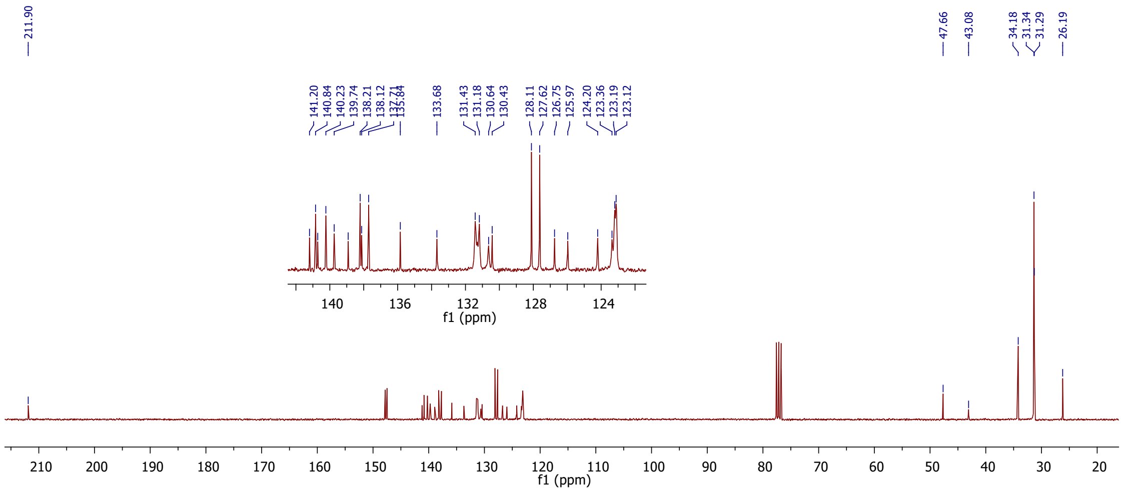

1. DEPT, 75MHz, $CDCl_3$, 298 K.

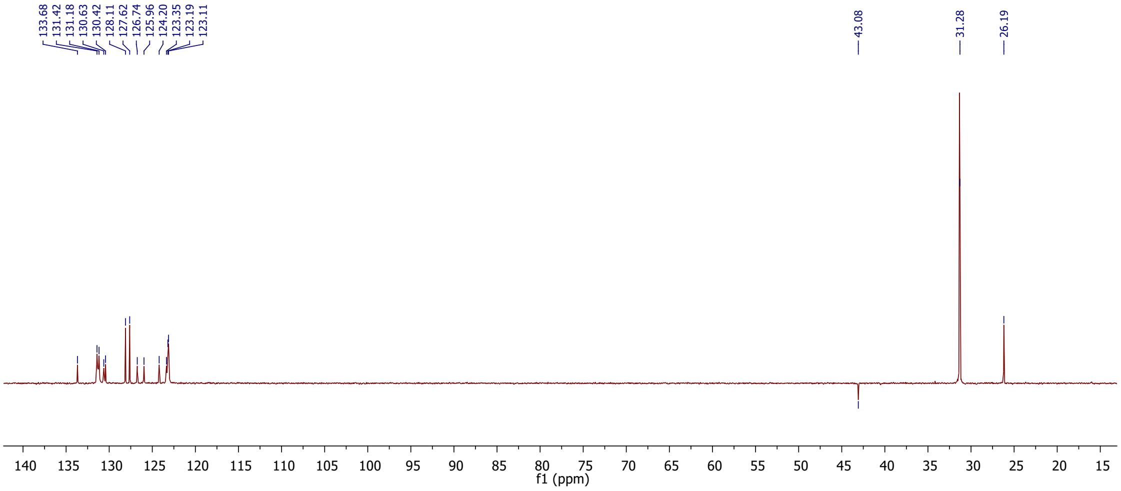

2. ^{1}H, 300MHz, $CDCl_3$, 298 K.

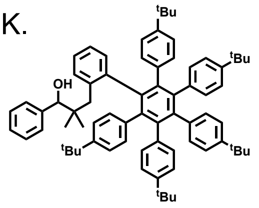

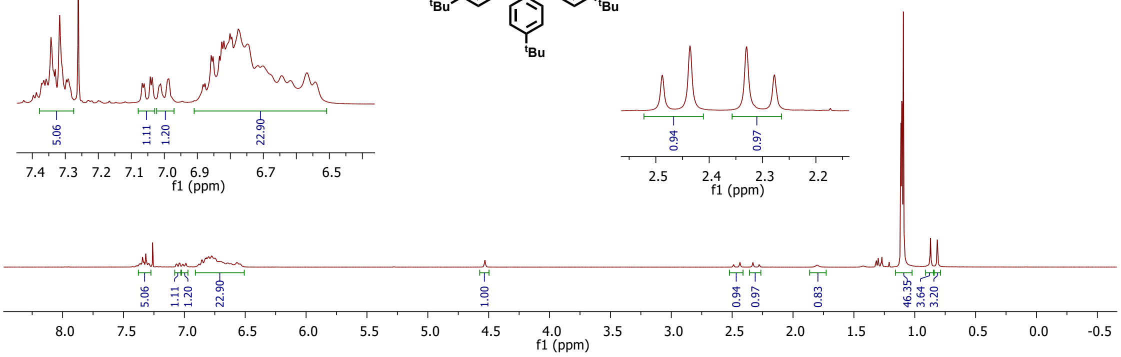

2. ^{13}C {^{1}H},75MHz, $CDCl_3$, 298 K.

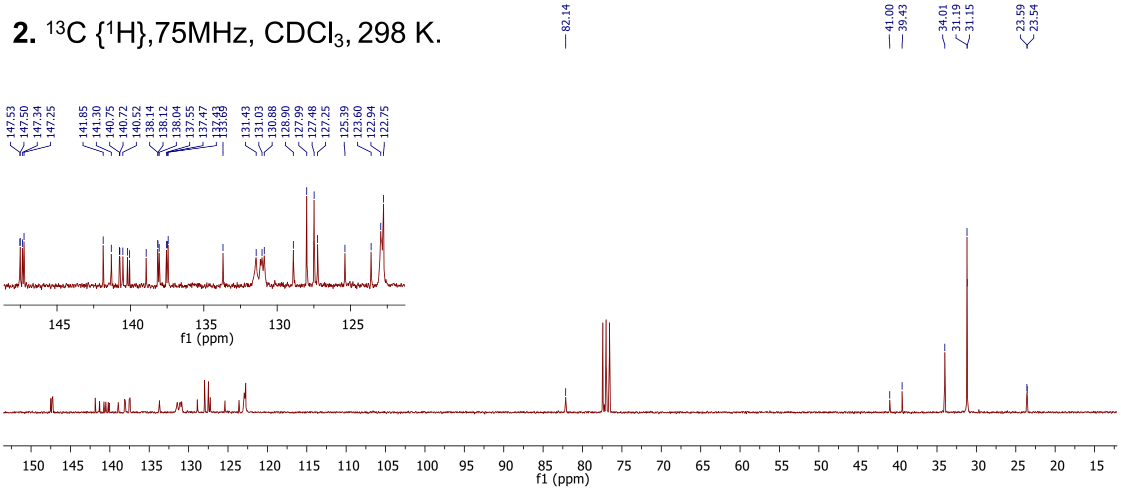

2. DEPT, 75MHz, $CDCl_3$, 298 K.

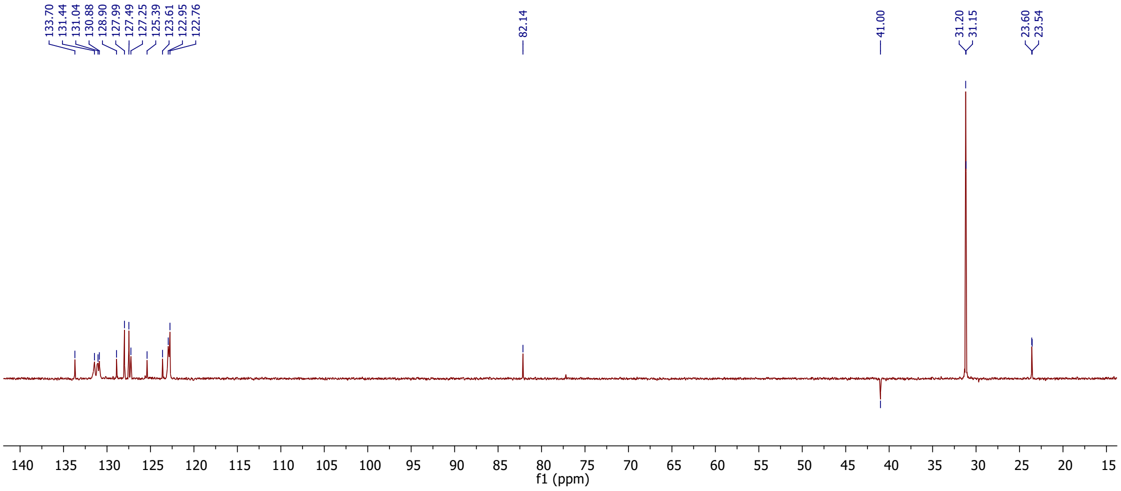

3. ^{1}H, 300MHz, $CDCl_3$, 298 K.

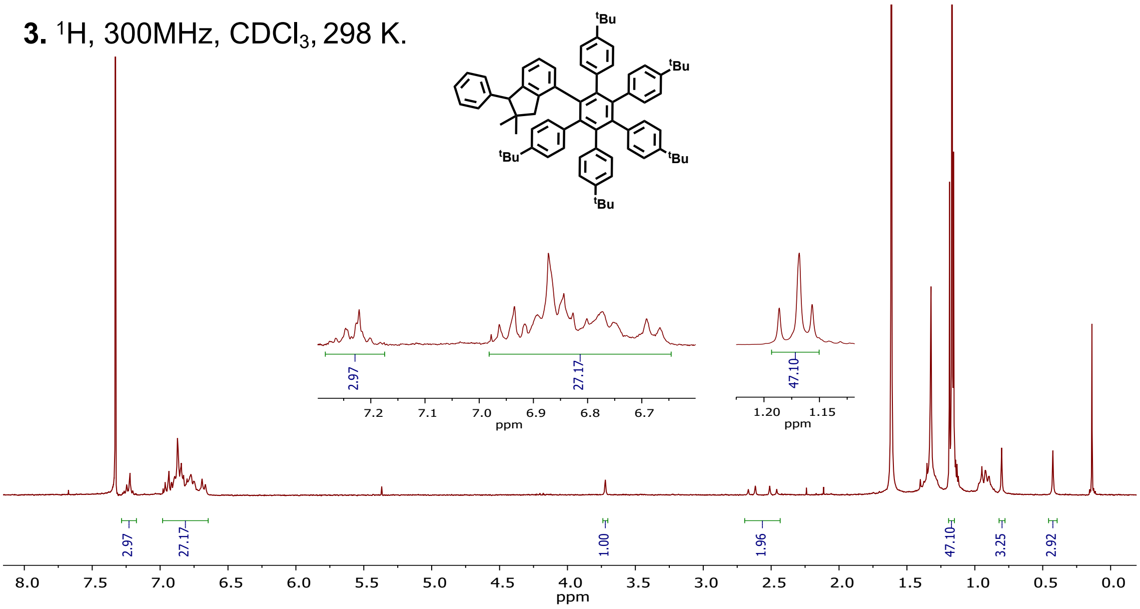

3. ^{13}C {^{1}H},75MHz, $CDCl_3$, 298 K.

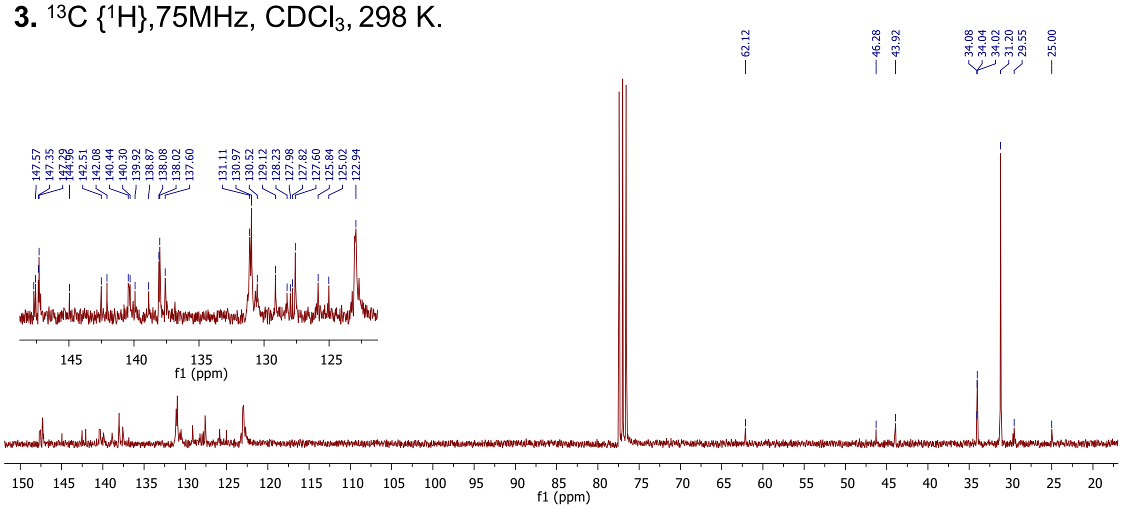

3. DEPT, 75MHz, $CDCl_3$, 298 K.

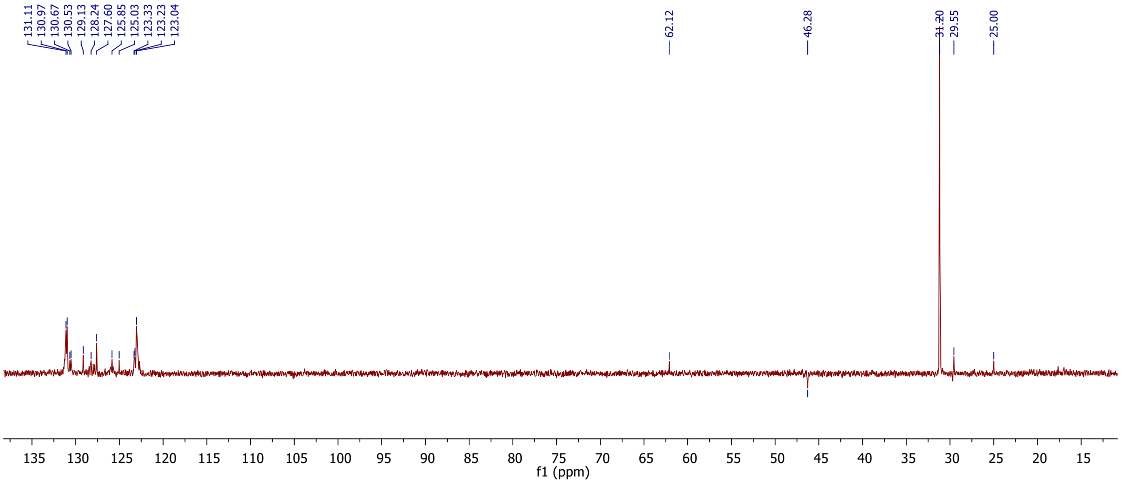

4. ^{1}H, 300MHz, $CDCl_3$, 298 K.

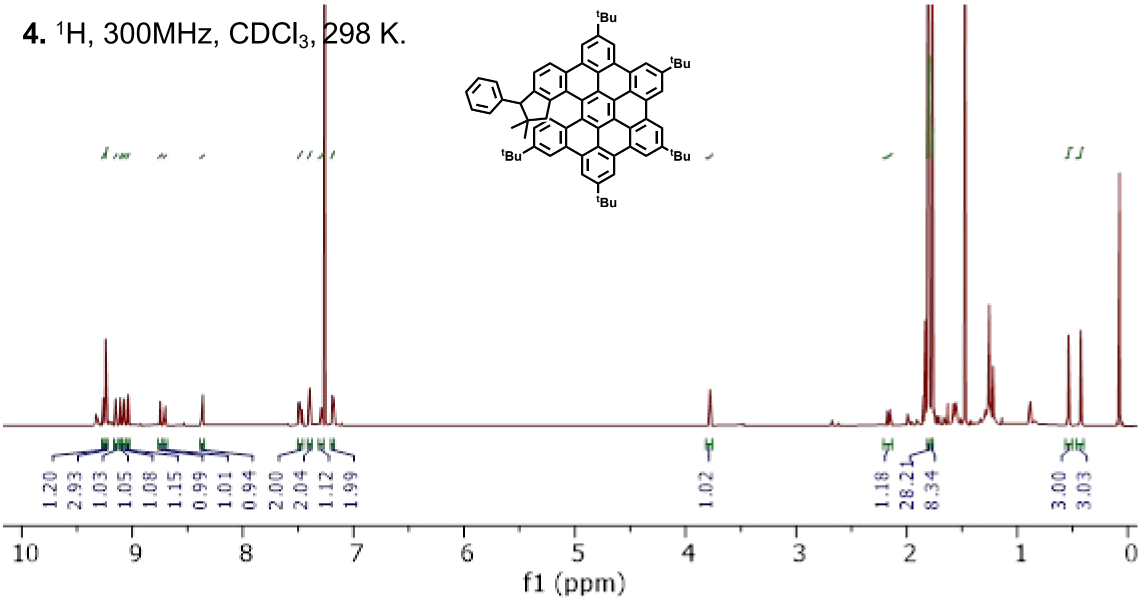

4. ^{13}C {^{1}H},75MHz, $CDCl_3$, 298 K.

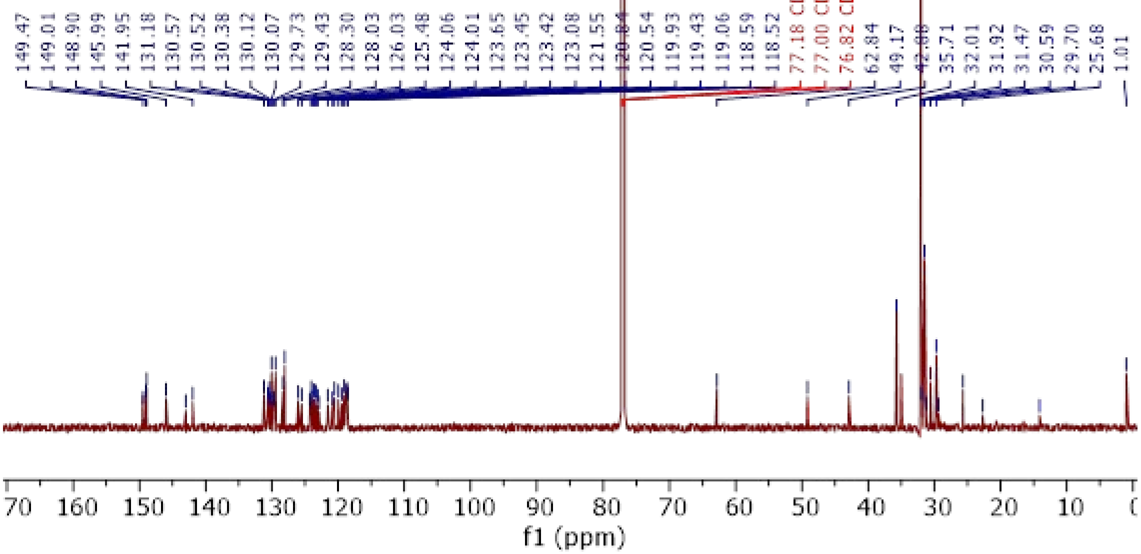

4. DEPT, 75MHz, $CDCl_3$, 298 K.

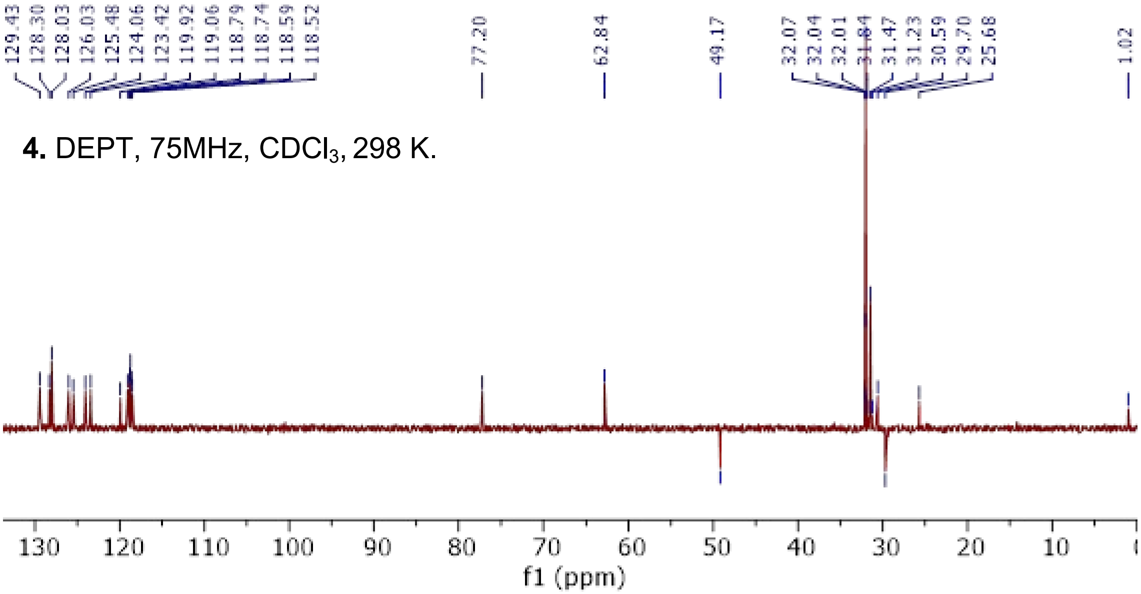

2D-NMR

HSQC

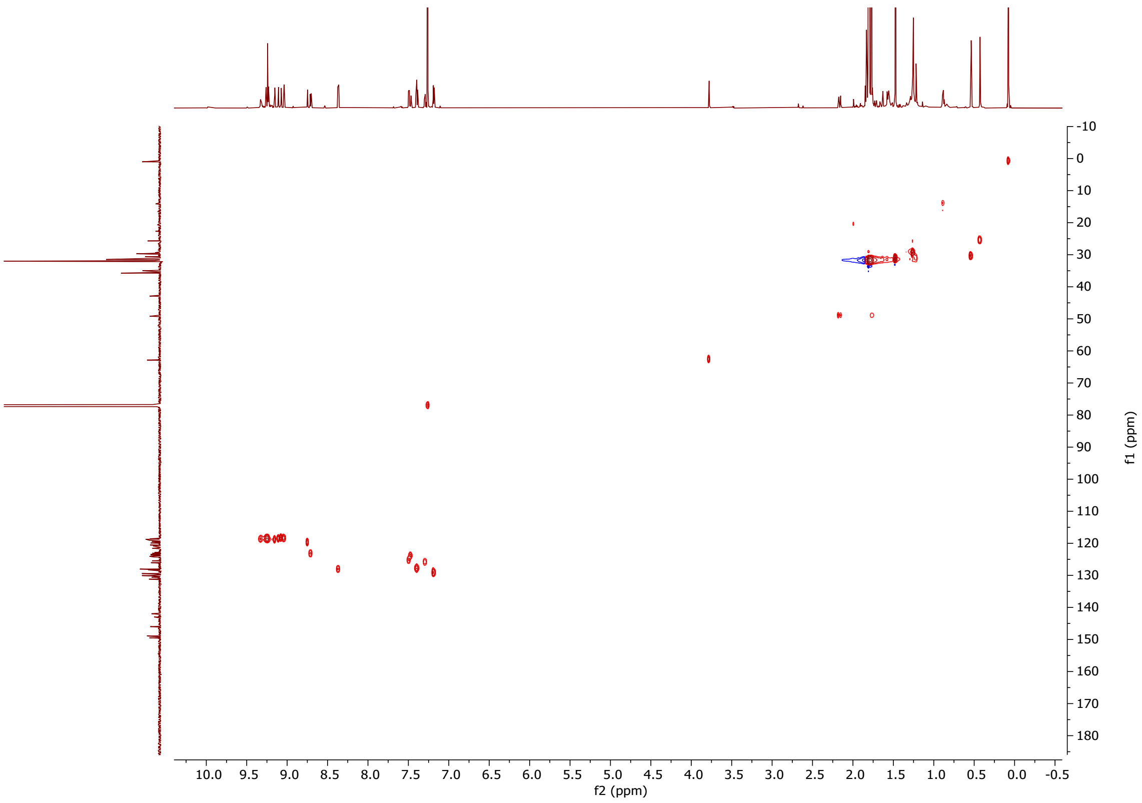

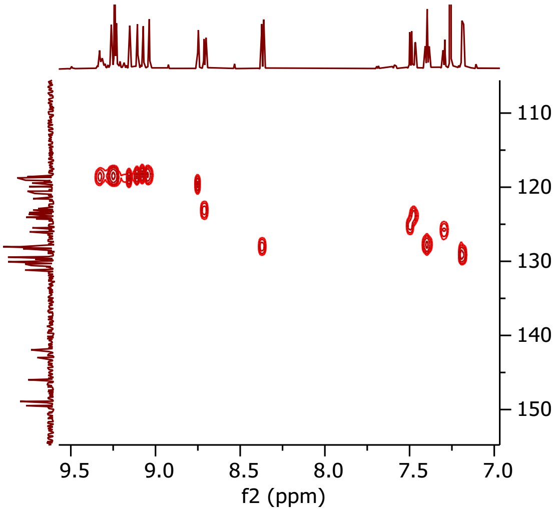

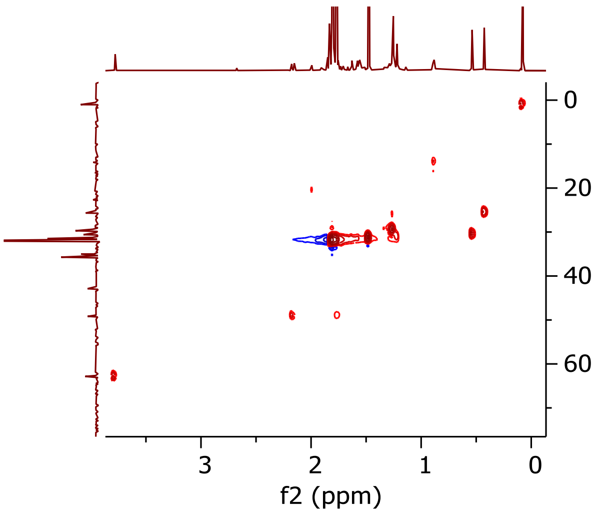

HMBC

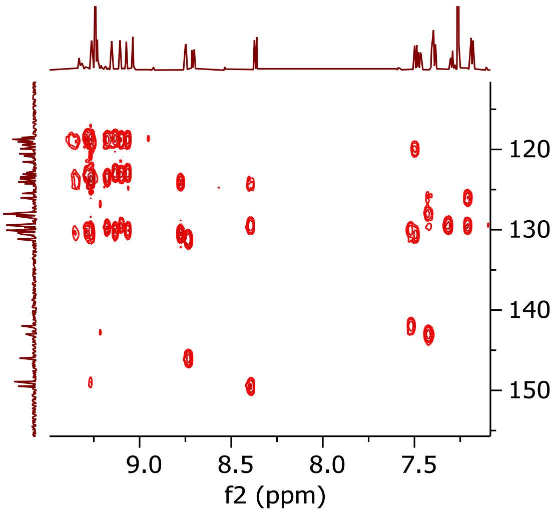
120
130
140
150
9.0
8.5
8.0
7.5
f2 (ppm)

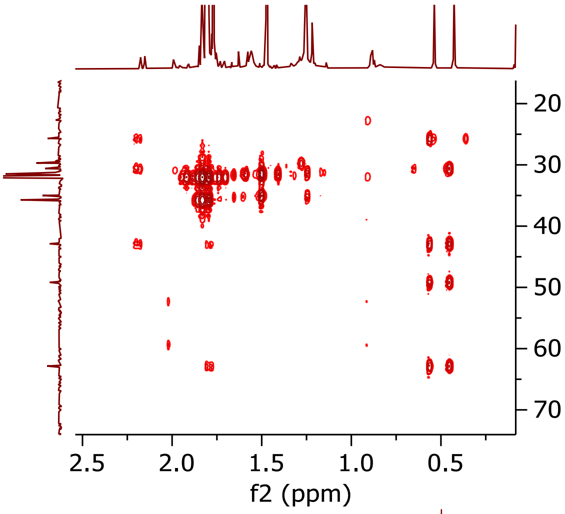
20
30
40
50
60
70
2.5
2.0
1.5
1.0
0.5
f2 (ppm)

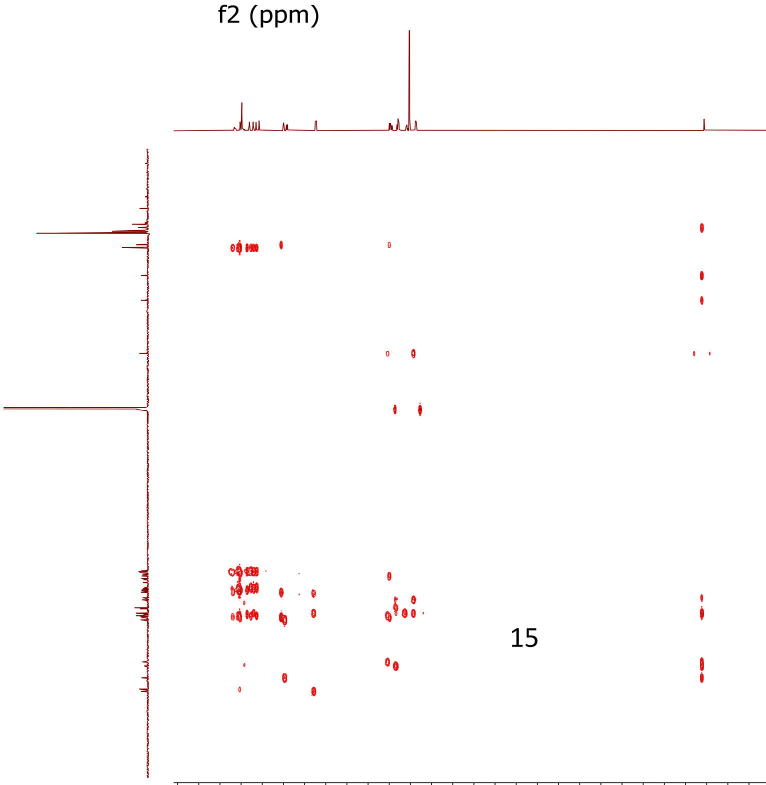

COSY

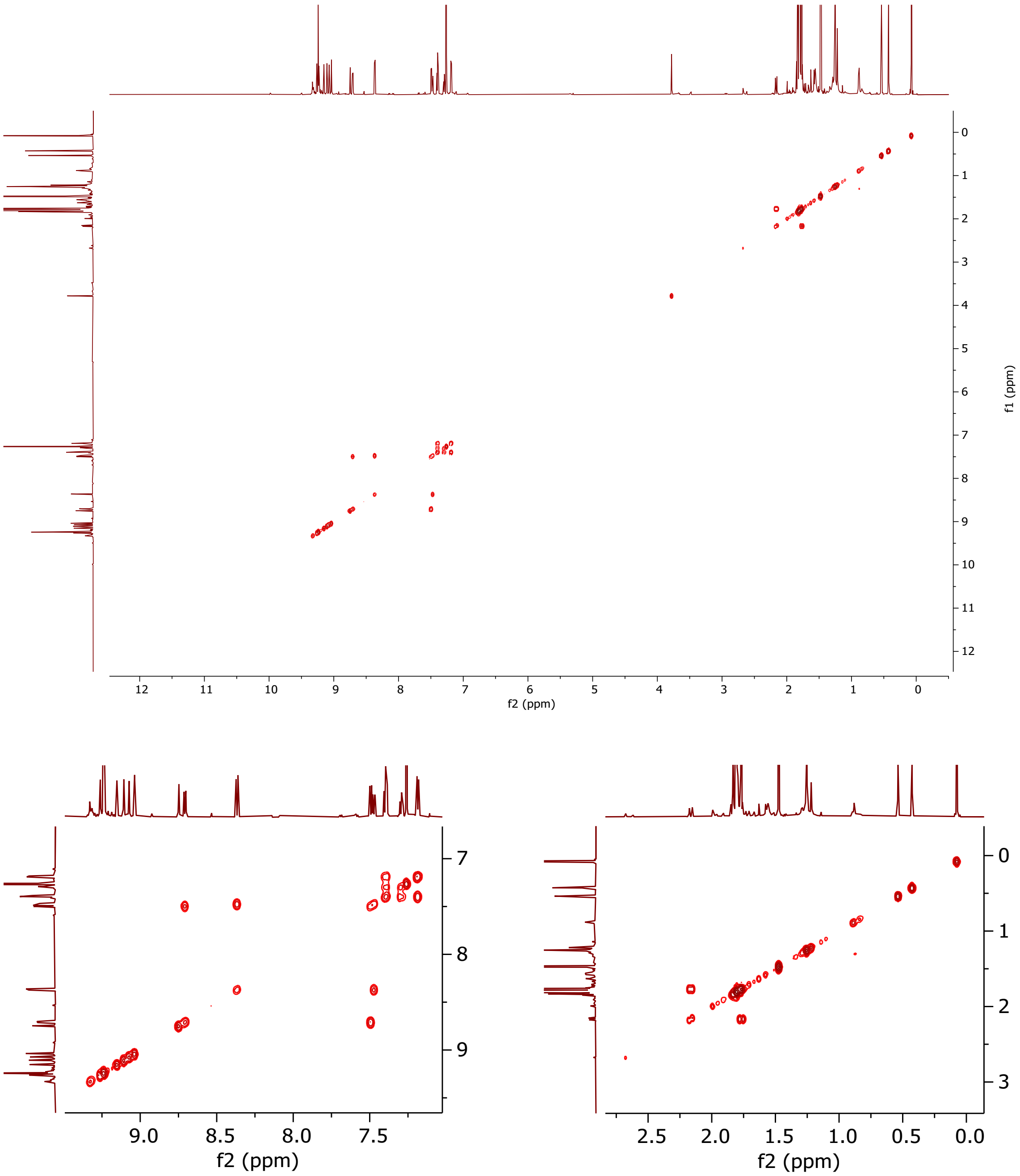

^{1}H ROESY

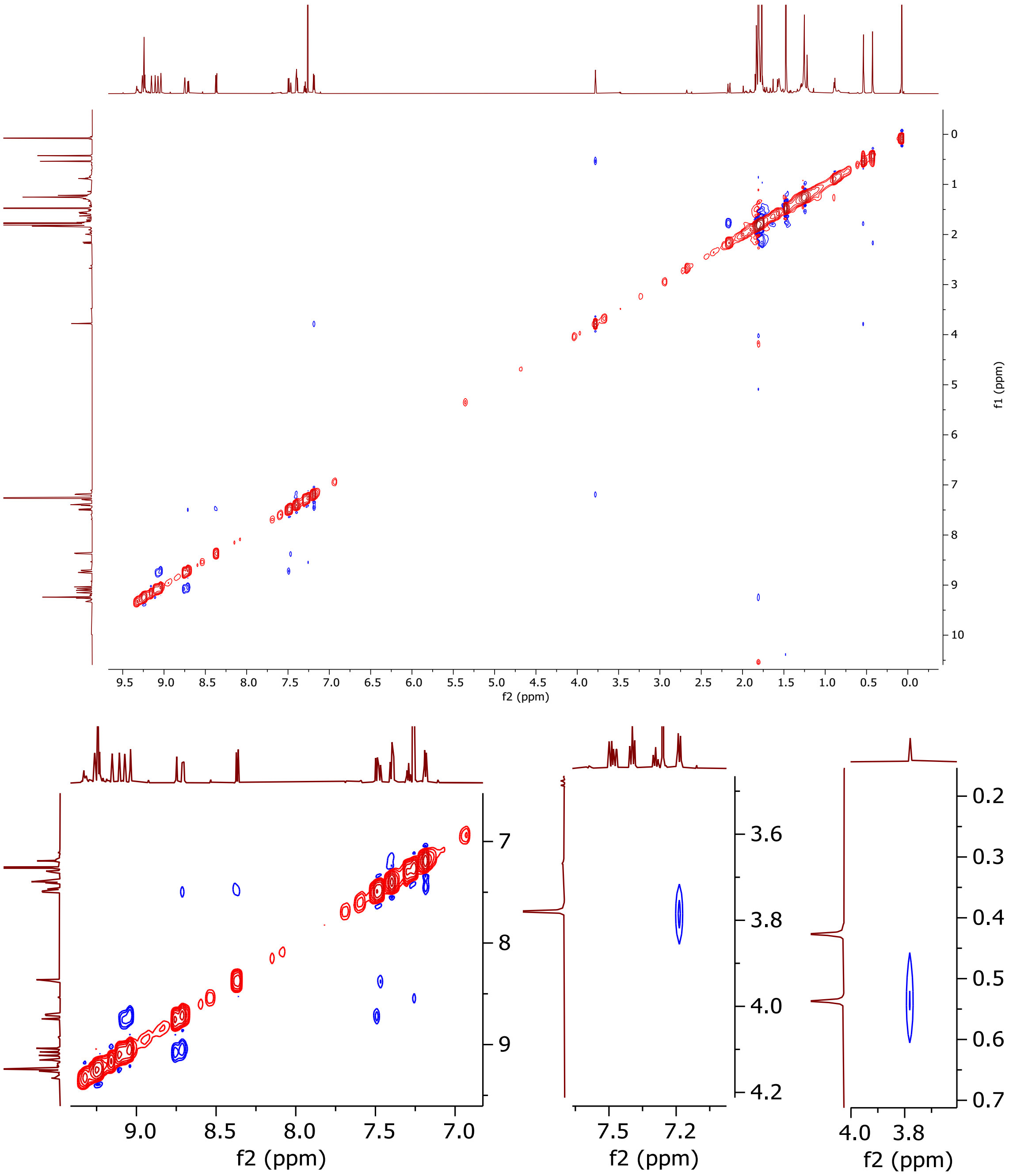

f1 (ppm)
f2 (ppm)
9.5 9.0 8.5 8.0 7.5 7.0 6.5 6.0 5.5 5.0 4.5 4.0 3.5 3.0 2.5 2.0 1.5 1.0 0.5 0.0
0 1 2 3 4 5 6 7 8 9 10
9.0 8.5 8.0 7.5 7.0
7 8 9
7.5 7.2
3.6 3.8 4.0 4.2
4.0 3.8
0.2 0.3 0.4 0.5 0.6 0.7

^{1}H ROESY Experiment and Diastereomeric Assignment

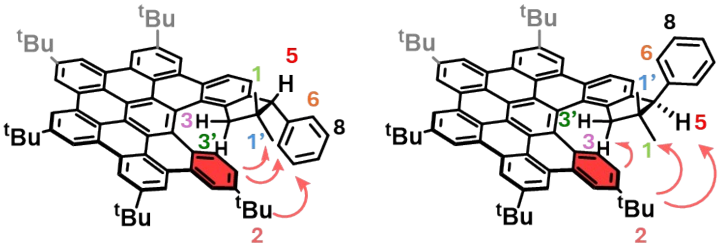

Figure S1: Pair of diastereomers of **4** and the expected key ROE correlation for each diastereomer.

The ^{1}H NMR assignment was carried out using 2D NMR experiments, with **^{1}H ROESY correlations** playing a key role in determining the diastereomeric configuration (**Figure S1**). The crucial through-space interactions observed in the ROESY spectrum are summarized as follows:

- Irradiation of proton **5 (Figure S3**, **δ 3.78 ppm)** showed a correlation with the closest **methyl group 1 (δ 0.54 ppm)**, indicating their spatial proximity.
- Irradiation of methyl **1 (Figure S4, δ 0.54 ppm)** correlated with proton **3 (δ 1.75 ppm)** but did **not** show correlation with **^{t}Bu 2 (δ 1.47 ppm)**, suggesting that **protons 1 and 2 are not close in space.** This observation supports the assignment of the **(S,M) diastereomer** as the most probable configuration.
- Irradiation of methyl **1' (Figure S5, δ 0.42 ppm)** exhibited a correlation with **^{t}Bu 2 and proton 6**, confirming its proximity to both **^{t}Bu 2** and the **terminal phenyl group,** further supporting the **(*S*,*M*) diastereomer** assignment.
- Irradiation of **^{t}Bu 2 (Figure S6, δ 1.47 ppm)** correlated with methyl **1' (δ 0.42 ppm)** but **not** with methyl **1**, reinforcing the proposed diastereomeric structure.
- Final validation of the **(*S*,*M*) diastereomer** was confirmed by the observed correlation between **^{t}Bu 2 (δ 1.47 ppm)** and **proton 8 (δ 7.39 ppm) (Figure S6)**.

These ROESY interactions provide conclusive evidence for the spatial arrangement of protons, ensuring the correct diastereomeric assignment of the molecule.

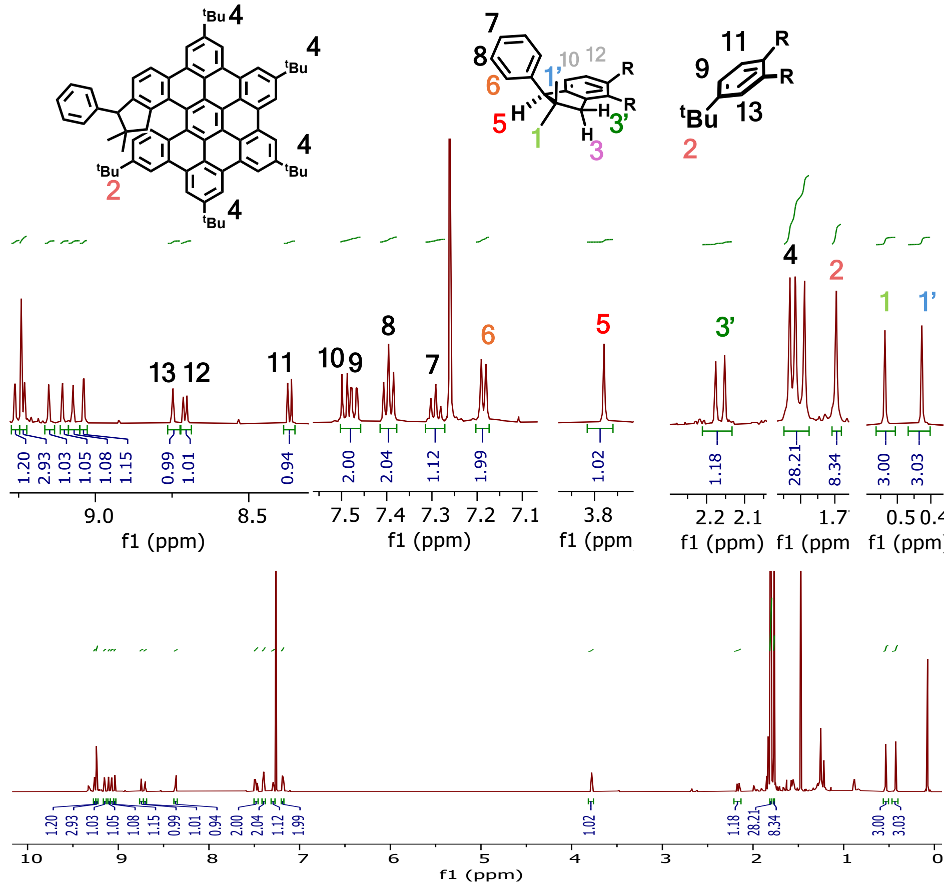

Figure S2: ^{1}H NMR signal assignment of **4**.

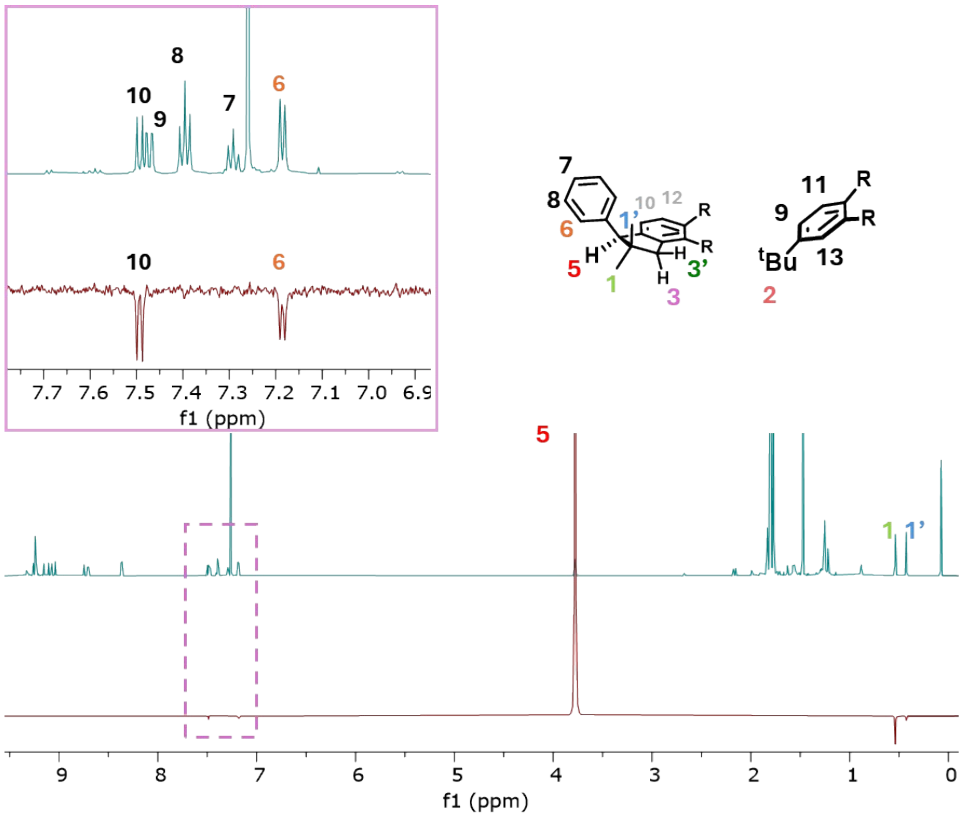

Figure S3: 1D ROESY irradiating the proton **5** signal at 3.78 ppm.

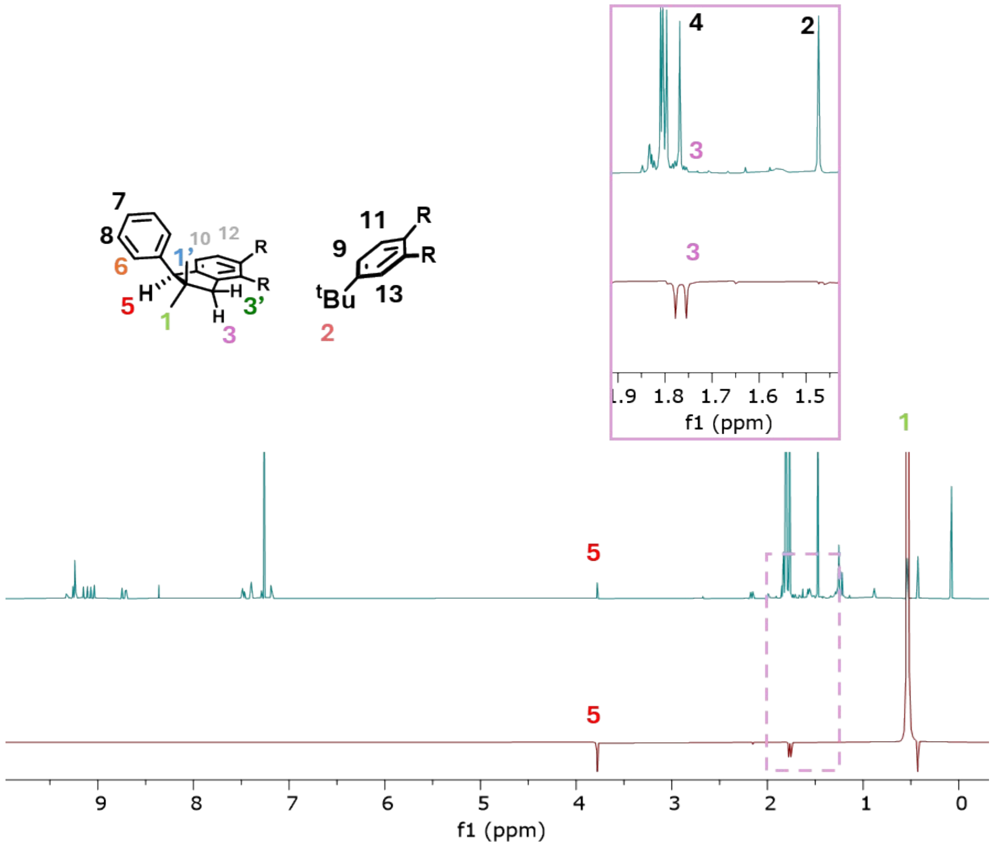

Figure S4: 1D ROESY irradiating the **methyl group 1** signal at 0.54 ppm.

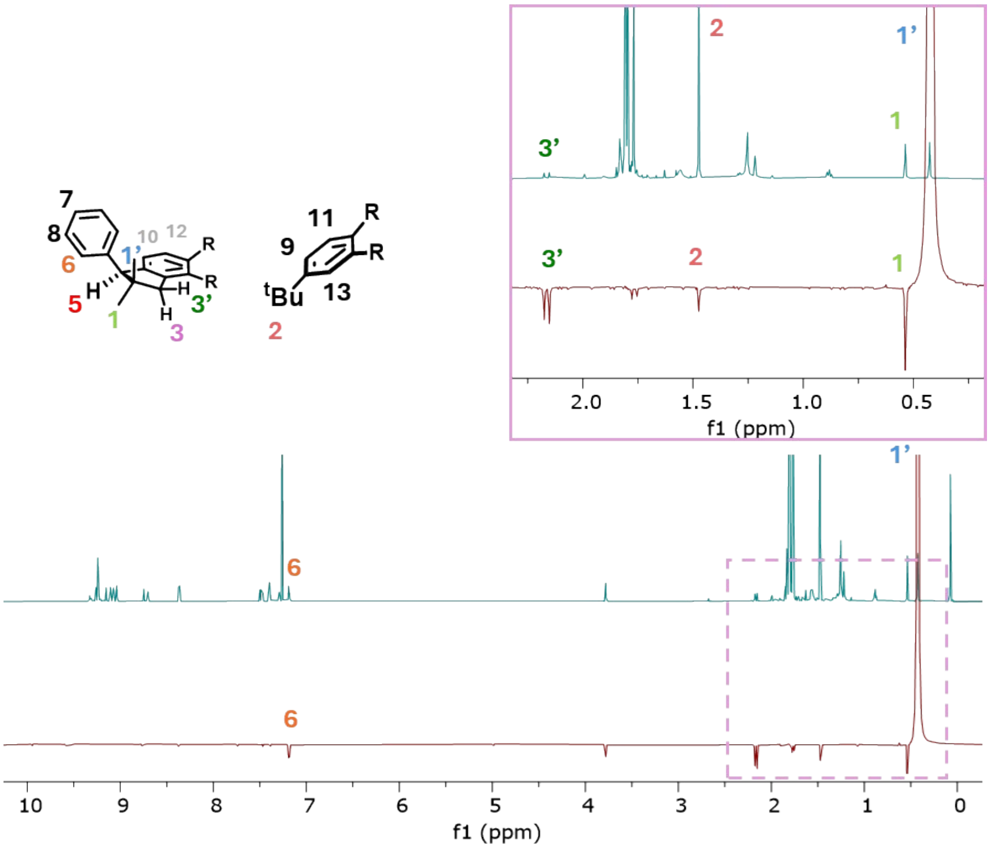

Figure S5: 1D ROESY irradiating methyl **1' signal** at 0.42 ppm.

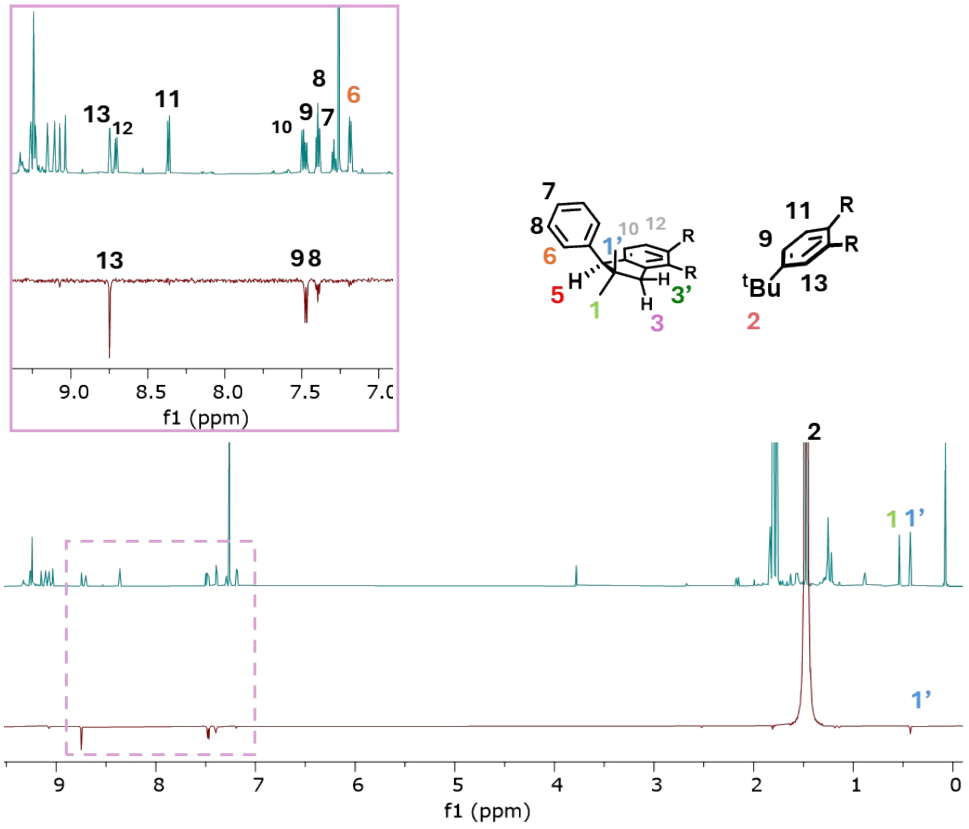

Figure S6: 1D ROESY irradiating ^{t}**Bu 2** signal at 1.47 ppm.

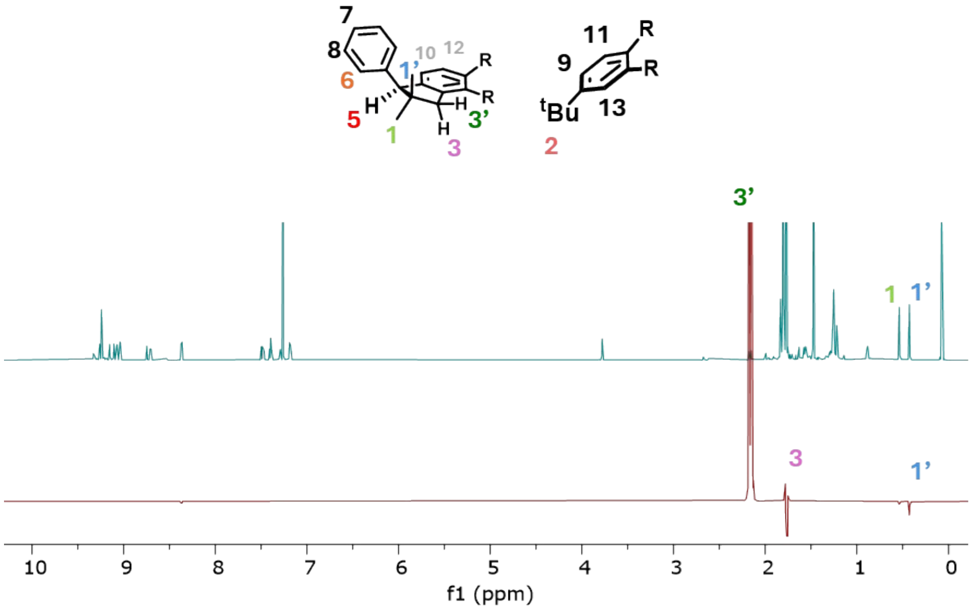

Figure S7: 1D ROESY irradiating proton **3’** signal at 2.16 ppm.

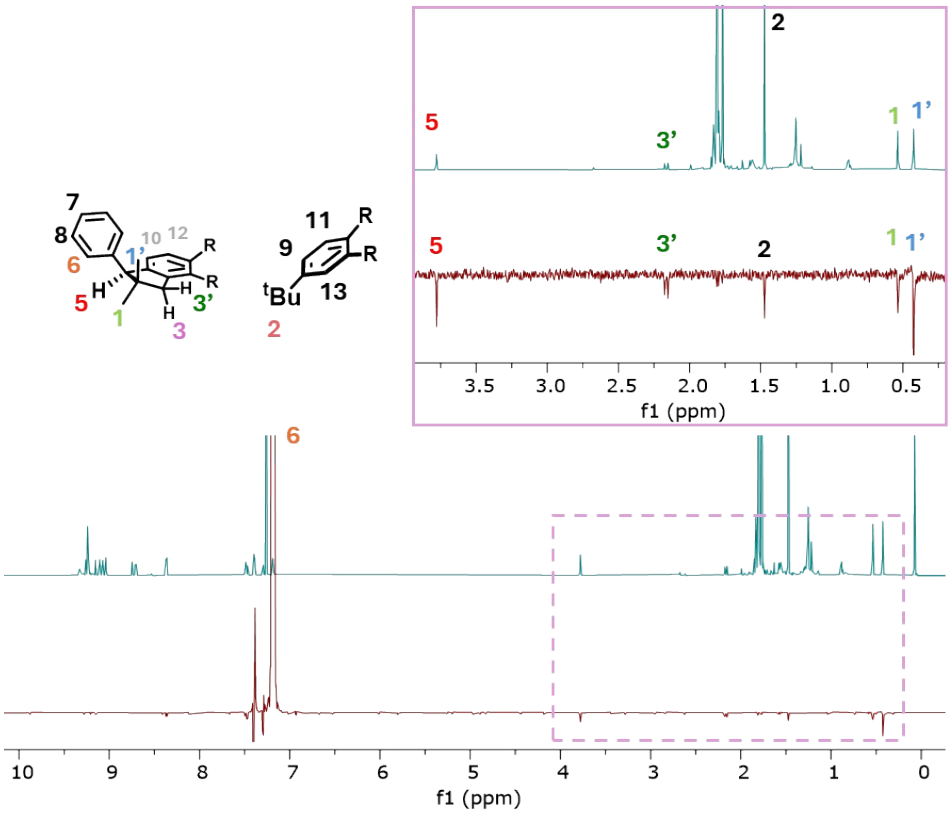

Figure S8: 1D ROESY irradiating **proton 6** signal at 7.18 ppm.

HPLC separation

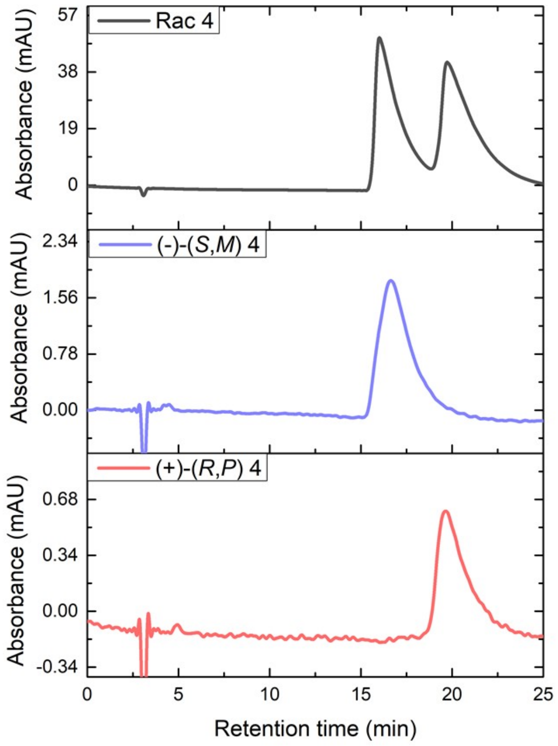

Figure S9: Chiral HPLC chromatograms for **4** racemic (top) and both enantiopure **(-)-(*S*,*M*) 4** (middle) and **(+)-(*R*,*P*) 4** (bottom). (Control Method: (*R*,*R*) ULMO (5 µm, 100 Å, 25 cm x 4.6 mm ID), Hex:DCM (95:5), 1 mL/min, 25°C, λ = 360 nm).

Chiral Dichroism and CPL espectra

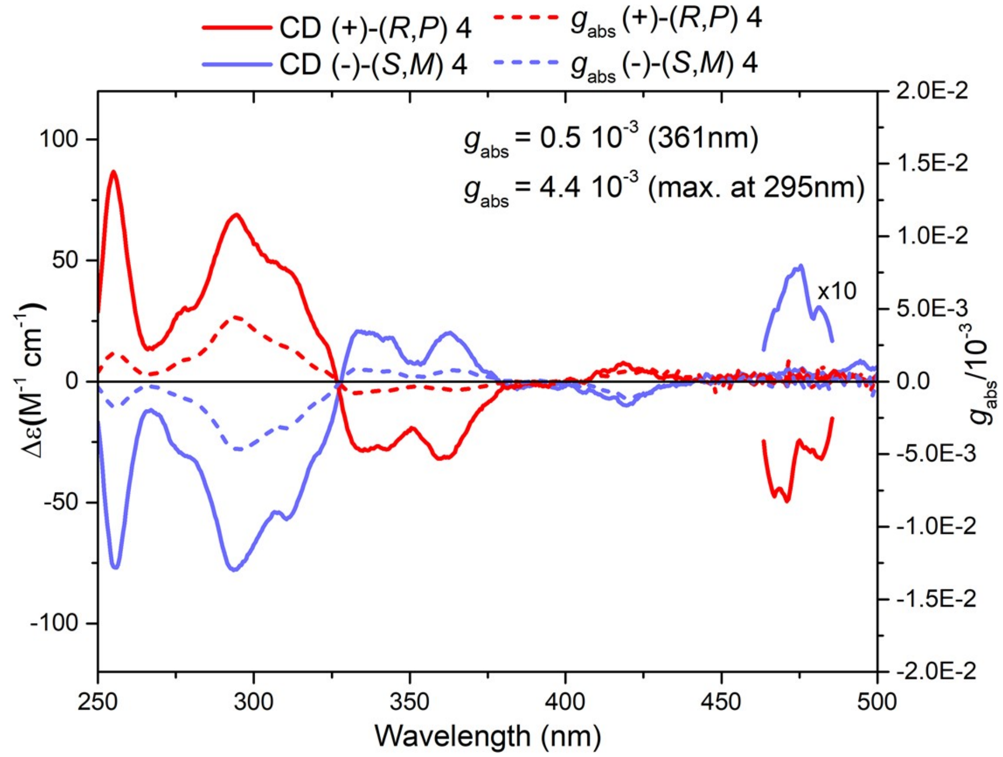

Figure S10: Circular Dichroism spectroscopic data and absorptive dissymmetry factor (g_{abs}) values of **(*R*,*P*)-4** and **(*S*,*M*)-4** (6 µM, $CHCl_3$, 20°C, 10 mm).

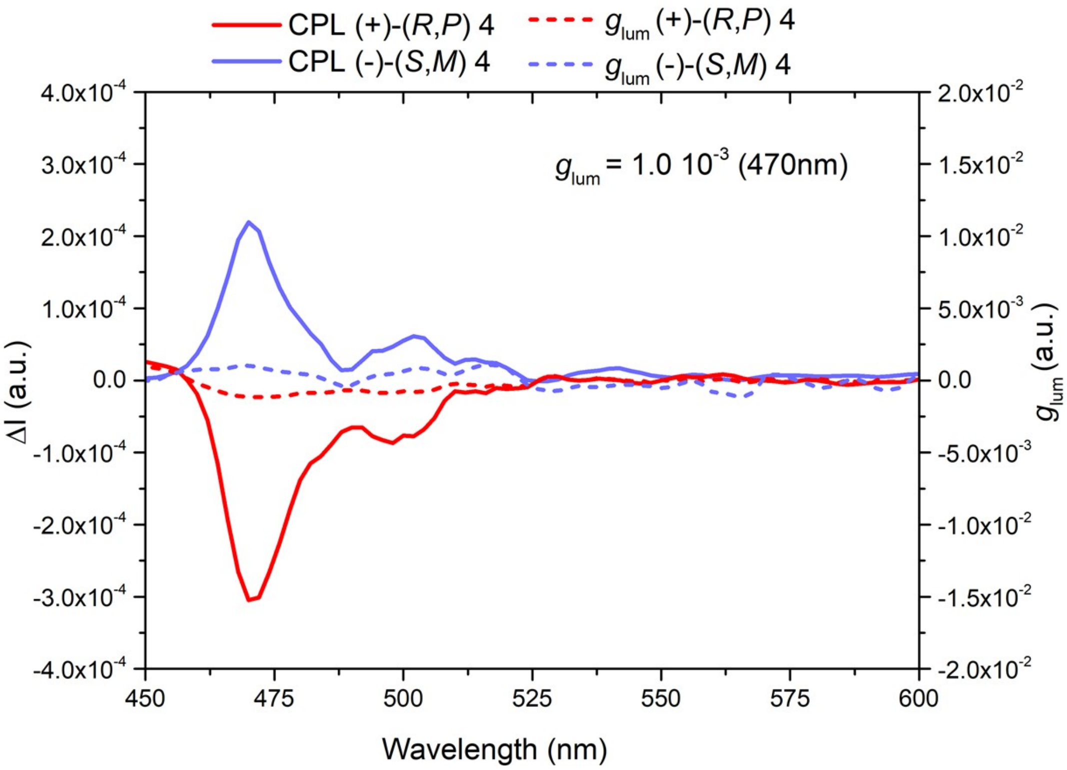

Figure S11: Circularly Polarized Luminescence spectroscopic data and luminescence dissymmetry factor (g_{lum}) values of **(*R*,*P*)-4** and **(*S*,*M*)-4** (ca. 2 µM, $CHCl_3$, 20°C, 10 mm).

Photophysical Studies

Molar extinction coefficient

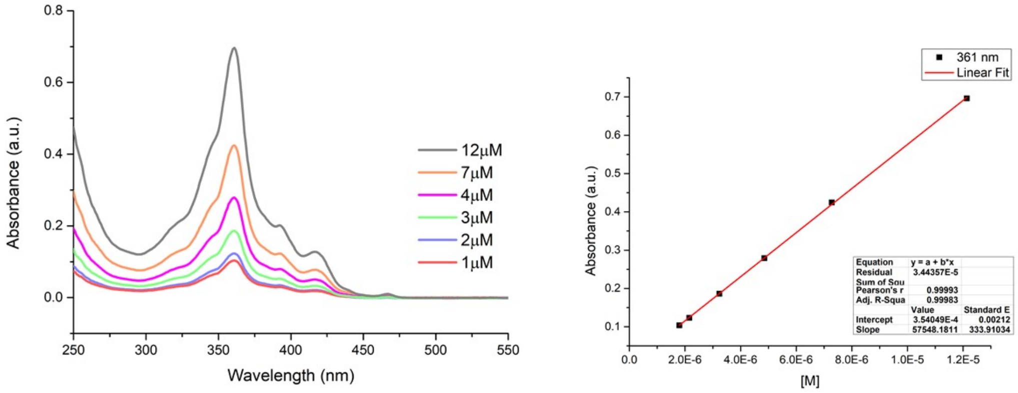

Figure S12: Calculation of molar extinction coefficient of **4** through Beer-Lambert law.

Fluorescence quantum yield calculation for 4

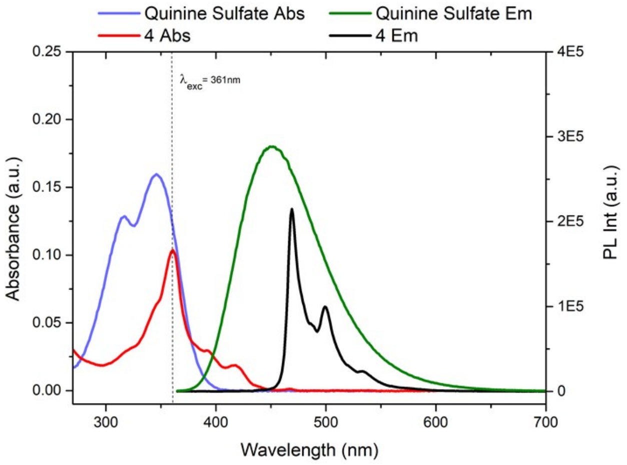

Figure S13: Absorption and emission spectra of quinine sulfate (reference, R) and **4** (sample, S) for fluorescence quantum yield calculation of **4**.

$$\Phi_F = \Phi_R \times \left(\frac{\eta_S}{\eta_R}\right)^2 \times \frac{1-10^{-Abs_R}}{1-10^{-Abs_S}} \times \frac{Int_S}{Int_R}$$

$$\Phi_F = 0.55 \times \left(\frac{1.443}{1.333}\right)^2 \times \frac{1-10^{-0.12431}}{1-10^{-0.10468}} \times \frac{6421021}{27678842}$$

$$\Phi_F = 0.17 = 17\%$$

Brightness (*B*) and CPL Brightness (B_{CPL}) calculation for 4

$$B = \varepsilon_{(361\ nm)} \times \Phi_F = 57548\ M^{-1}cm^{-1}\ 0.17 = 9783\ M^{-1}\ cm^{-1}$$

$$B_{CPL} = \varepsilon_{(361\ nm)} \times \Phi_F \times \frac{g_{lum}}{2} = B \times \frac{g_{lum}}{2} = 9783\ M^{-1}\ cm^{-1} \times \frac{1.0 \times 10^{-3}}{2}$$

$$B_{CPL} = 9783\ M^{-1}\ cm^{-1} \times \frac{1.0 \times 10^{-3}}{2} = 4.9\ M^{-1}\ cm^{-1}$$

Molecule	ε /M^{-1} cm^{-1} (λ_{abs}^{max}/nm)	Φ_F	*B* / M^{-1} cm^{-1}	$\|g_{abs}\|$ / 10^{-3}	$\|g_{lum}\|$ / 10^{-3}	B_{CPL} / M^{-1} cm^{-1}
**4**	57548 (361)	0.17	9783	4.4	1.0	4.9
**^{t}Bu-HBC**	141700 (360)	0.04	5668	-	-	-
**[5]-helicene**	43500 (356)	0.057	2680	7.6	2.7	3.3

Table 1: Compilation of the photophysical properties of **4**, ^{t}Bu-HBC and [5]-helicene.

Computational Details

All the geometry optimizations and frequency calculations reported in this paper were obtained with the ORCA 6.0.1 program.[3] Electron correlation was partially taken into account using the B3LYP[4] functional in conjunction with the D3(BJ) dispersion correction suggested by Grimme et al.[5], the resolution-of-identity,[6] RI(JCOSX), approach and the double-ζ quality plus polarization functions def2-SVP[7] basis set for all atoms. All species were characterized by frequency calculations and have positive definite Hessian matrices. Solvent effects were taken into account by using the conductor-like polarizable continuum model (CPCM).[8] This level is denoted CPCM-RI-B3LYP-D3BJ/def2-SVP, which was proven to provide good results for strongly related systems.[9] Non-covalent interactions were visualized by means of the NCIPlot program.[10] Nuclear Independent Chemical Shift (NICS)[11] values were computed using the gauge invariant atomic orbital (GIAO) method[12] at the B3LYP/def2-SVP//CPCM-RI-B3LYP-D3BJ/def2-SVP level. Calculations of the absorption spectrum were accomplished using time-dependent density functional theory (TD-DFT)[13] at the same PCM-B3LYP-D3/def2-SVP//CPCM-RI-B3LYP-D3BJ/def2-SVP level. Both the NICS and TD-DFT calculations were calculated using the Gaussian 09 program.[14]

---

[3] F. Neese, *Rev. Comput. Mol. Sci.*, 2012, **2**, 73.

[4] a) A. D. Becke, *J. Chem. Phys.*, 1993, **98**, 5648; b) C. Lee, W. Yang and R. G. Parr, *Phys. Rev. B,* 1998, **37**, 785; c) S. H. Vosko, L. Wilk and M. Nusair, *Can. J. Phys.*, 1980, **58**, 1200.

[5] S. Grimme, J. Antony, S. Ehrlich and H. Krieg, *J. Chem. Phys.*, 2010, **132**, 154104.

[6] K. Eichkorn, O. Treutler, H. Öhm, M. Häser and R. Ahlrichs, *Chem. Phys. Lett.,* 1995, **242**, 652.

[7] F. Weigend and R. Alhrichs, *Phys. Chem. Chem. Phys*., 2005, **7**, 3297.

[8] V. Barone and M. Cossi, *J. Phys. Chem. A,* 1998, **102**, 1995.

[9] See, for instance: a) Z. Zhou, J. M. Fernández-García, Y. Zhu, P. J. Evans, R. Rodríguez, J. Crassous, Z. Wei, I. Fernández, M. A. Petrukina and N. Martin, *Angew. Chem. Int. Ed*., 2022, **61**, e202115747; b) Z. Zhou, Y. Zhu, J. M. Fernández-García, Z. Wei, I. Fernández, M. A. Petrukhina and N. Martín, *Chem. Commun*., 2022, **58**, 5574.

[10] E. R. Johnson, S. Keinan, P. Mori-Sánchez, J. Contreras-García, A. J. Cohen and W. Yang, W. *J. Am. Chem. Soc*., 2010, **132**, 6498.

[11] Z. Chen, C. S. Wannere, C. Corminboeuf, R. Puchta and P. von R. Schleyer, *Chem. Rev*., 2005, **105**, 3842.

[12] K. Wolinski, J. F. Hilton and P. Pulay, *J. Am. Chem. Soc*., 1990, **112**, 8251.

[13] a) M. E. Casida, *Recent Developments and Applications of Modern Density Functional Theory*; Elsevier: Amsterdam, 1996; Vol. 4; b) M. E. Casida, D. P. Chong, *Recent Advances in Density Functional Methods*; World Scientific: Singapore, 1995; Vol. 1, p 155.

[14] Gaussian 09, Revision *E.01*, M. J. Frisch, G. W. Trucks, H. B. Schlegel, G. E. Scuseria, M. A. Robb, J. R. Cheeseman, G. Scalmani, V. Barone, B. Mennucci, G. A. Petersson, H. Nakatsuji, M. Caricato, X. Li, H. P. Hratchian, A. F. Izmaylov, J. Bloino, G. Zheng, J. L. Sonnenberg, M. Hada, M. Ehara, K. Toyota, R. Fukuda, J. Hasegawa, M. Ishida, T. Nakajima, Y. Honda, O. Kitao, H. Nakai, T. Vreven, J. A. Montgomery, Jr., J. E. Peralta, F. Ogliaro, M. Bearpark, J. J. Heyd, E. Brothers, K. N. Kudin, V. N. Staroverov, R. Kobayashi, J. Normand, K. Raghavachari, A. Rendell, J. C. Burant, S. S. Iyengar, J. Tomasi, M. Cossi, N. Rega, J. M. Millam, M. Klene, J. E. Knox, J. B. Cross, V. Bakken, C. Adamo, J. Jaramillo, R. Gomperts, R. E. Stratmann, O. Yazyev, A. J. Austin, R. Cammi, C. Pomelli, J. W. Ochterski, R. L. Martin, K. Morokuma, V. G. Zakrzewski, G. A. Voth, P. Salvador, J. J. Dannenberg, S. Dapprich, A. D. Daniels, O. Farkas, J. B. Foresman, J. V. Ortiz, J. Cioslowski, and D. J. Fox, Gaussian, Inc., Wallingford CT, 2009.

Cartesian coordinates (in Å) and free energies (in a.u., at 195 K) of all the stationary points discussed in the text. All calculations have been performed at the CPCM-RI-B3LYP-D3BJ/def2-SVP level.

[INT1(*S*)]⁺: G= -2826.81727041

C	0.150003000	0.247046000	-0.461762000
C	1.537102000	0.451510000	-0.331446000
C	2.024242000	1.729281000	0.029580000
C	1.129703000	2.791265000	0.275758000
C	-0.259896000	2.581408000	0.166118000
C	-0.737708000	1.308077000	-0.199466000
C	2.496655000	-0.665391000	-0.561798000
C	2.578503000	-1.325988000	-1.791954000
C	3.533064000	-2.324175000	-2.011207000
H	3.564780000	-2.799393000	-2.991438000
C	4.438395000	-2.703735000	-1.010093000
C	4.334485000	-2.048378000	0.230165000
C	3.386858000	-1.055347000	0.451870000
C	3.494896000	1.947257000	0.137302000
C	4.326756000	1.766378000	-0.977602000
C	5.707853000	1.899538000	-0.866613000
H	6.321015000	1.742472000	-1.756559000
C	6.321308000	2.215387000	0.359736000
C	5.479371000	2.418846000	1.463250000
H	5.899664000	2.671467000	2.436586000
C	4.091876000	2.291437000	1.354172000
C	-2.200833000	1.061589000	-0.300138000
C	-3.026096000	1.774778000	-1.130845000
C	-4.469881000	1.522382000	-1.188854000
H	-4.785261000	1.394582000	-2.245356000
C	-5.035698000	0.449553000	-0.351814000
C	-4.171109000	-0.236501000	0.486685000
H	-4.542562000	-1.019622000	1.146225000
C	-2.806284000	0.064571000	0.509893000
H	-2.171687000	-0.481935000	1.209996000
C	-0.397577000	-1.056141000	-0.943527000
C	-0.951923000	-1.129753000	-2.234481000
C	-1.465335000	-2.327868000	-2.735355000
H	-1.879868000	-2.357904000	-3.746174000
C	-1.445667000	-3.487210000	-1.949898000
H	-1.854929000	-4.425776000	-2.332842000
C	-0.907026000	-3.420219000	-0.667238000
C	-0.387520000	-2.218870000	-0.162591000
C	-0.844322000	-4.516134000	0.373715000
H	-0.464294000	-5.454200000	-0.060017000
C	0.223839000	-3.952889000	1.396064000
C	0.078935000	-2.412276000	1.255448000
H	1.016544000	-1.887106000	1.485465000
H	-0.682639000	-2.028306000	1.959334000
H	-0.967181000	-0.230774000	-2.855559000
H	1.903880000	-1.043837000	-2.601850000

H	5.008604000	-2.316910000	1.046104000
H	3.342616000	-0.562470000	1.424460000
H	3.466593000	2.444860000	2.236261000
H	3.884644000	1.501898000	-1.940324000
H	-2.625807000	2.561708000	-1.772782000
H	-4.996109000	2.472709000	-0.939778000
C	5.518713000	-3.773220000	-1.218894000
C	-6.508182000	0.100992000	-0.425091000
C	5.461597000	-4.387402000	-2.625827000
H	6.247122000	-5.152427000	-2.727866000
H	4.492572000	-4.874094000	-2.818168000
H	5.628008000	-3.631244000	-3.408945000
C	6.906894000	-3.124995000	-1.023502000
H	7.023232000	-2.709768000	-0.010769000
H	7.703104000	-3.871934000	-1.176384000
H	7.059870000	-2.305935000	-1.744673000
C	5.326937000	-4.904186000	-0.186056000
H	4.343590000	-5.385025000	-0.309913000
H	6.104881000	-5.674266000	-0.315497000
H	5.392815000	-4.529513000	0.846586000
C	-7.309264000	1.086804000	-1.288649000
H	-6.965969000	1.097333000	-2.334151000
H	-8.366748000	0.784893000	-1.290943000
H	-7.253719000	2.113270000	-0.893647000
C	-7.106938000	0.076176000	1.000232000
H	-8.175905000	-0.177016000	0.934722000
H	-6.621181000	-0.674798000	1.639320000
H	-7.012641000	1.060862000	1.483590000
C	-6.600039000	-1.316131000	-1.052535000
H	-6.080348000	-2.065724000	-0.437759000
H	-7.660097000	-1.603469000	-1.130095000
H	-6.161121000	-1.328201000	-2.062030000
C	1.645644000	4.137030000	0.654228000
C	2.447951000	4.879175000	-0.224947000
C	1.333030000	4.702291000	1.894961000
C	2.910931000	6.144151000	0.128813000
C	1.805613000	5.968751000	2.249386000
C	2.602780000	6.721742000	1.374737000
H	2.707732000	4.460469000	-1.199514000
H	0.711371000	4.144150000	2.598411000
H	3.526398000	6.693336000	-0.587054000
H	1.539130000	6.363984000	3.229456000
C	-1.220724000	3.696057000	0.401500000
C	-1.209023000	4.836883000	-0.416321000
C	-2.180319000	3.627894000	1.418612000
C	-2.129860000	5.862718000	-0.221999000
C	-3.099840000	4.662252000	1.613535000
C	-3.100034000	5.802327000	0.796450000
H	-0.470525000	4.917334000	-1.216485000
H	-2.209510000	2.754207000	2.073978000
H	-2.089451000	6.730662000	-0.883429000
H	-3.822836000	4.563712000	2.422956000
C	-2.204557000	-4.819831000	0.978389000
C	-2.499226000	-6.114644000	1.431606000
C	-3.183485000	-3.826146000	1.129617000
C	-3.729004000	-6.406038000	2.029287000
H	-1.752589000	-6.904867000	1.313412000

C	-4.414475000	-4.110042000	1.726989000
H	-2.979613000	-2.821046000	0.761517000
C	-4.692292000	-5.403310000	2.181904000
H	-3.937946000	-7.422676000	2.372800000
H	-5.163011000	-3.319529000	1.831807000
H	-5.655546000	-5.629873000	2.645918000
C	-4.092322000	6.959197000	0.976598000
C	-5.094434000	6.693543000	2.110801000
H	-5.693057000	5.788637000	1.921257000
H	-4.590753000	6.575430000	3.082819000
H	-5.788638000	7.544019000	2.195073000
C	-3.307042000	8.246899000	1.308249000
H	-4.000689000	9.092521000	1.445021000
H	-2.728857000	8.122291000	2.237824000
H	-2.604209000	8.513043000	0.504130000
C	-4.883643000	7.160314000	-0.333472000
H	-4.220929000	7.400667000	-1.178422000
H	-5.449324000	6.250491000	-0.592146000
H	-5.600934000	7.989647000	-0.221916000
C	7.851194000	2.301246000	0.448013000
C	8.329668000	2.692417000	1.854708000
H	7.941651000	3.677660000	2.157616000
H	8.020694000	1.954049000	2.610907000
H	9.429484000	2.745426000	1.869342000
C	8.366885000	3.357233000	-0.552505000
H	9.465634000	3.427395000	-0.499858000
H	8.095779000	3.105160000	-1.588850000
H	7.948275000	4.350296000	-0.322467000
C	8.448175000	0.920764000	0.095809000
H	9.548474000	0.953662000	0.154349000
H	8.089092000	0.149942000	0.796688000
H	8.173044000	0.605525000	-0.922434000
C	3.132825000	8.120005000	1.721569000
C	2.705170000	8.570668000	3.126859000
H	1.609427000	8.625383000	3.222345000
H	3.084687000	7.891306000	3.906104000
H	3.109595000	9.574366000	3.330876000
C	4.675450000	8.108263000	1.661717000
H	5.089072000	7.382867000	2.380690000
H	5.042458000	7.840181000	0.659336000
H	5.074036000	9.105166000	1.911393000
C	2.585246000	9.136054000	0.696193000
H	2.898489000	8.887642000	-0.329252000
H	1.483755000	9.159027000	0.719656000
H	2.955061000	10.148504000	0.927039000
C	1.616896000	-4.373609000	0.901265000
H	2.404188000	-3.949842000	1.542545000
H	1.717518000	-5.470872000	0.910369000
H	1.803671000	-4.019398000	-0.123928000
C	0.025496000	-4.420014000	2.837255000
H	-0.953864000	-4.113502000	3.233991000
H	0.093754000	-5.517008000	2.914828000
H	0.806304000	-3.986496000	3.483306000

TS1(S-M)+: G = -2826.78541453 (i = -337 cm-1)

C	0.388986000	-0.107243000	-0.161620000

C	1.809823000	-0.153610000	-0.091048000
C	2.524134000	1.032612000	0.151470000
C	1.838998000	2.271038000	0.187151000
C	0.454427000	2.328137000	-0.050198000
C	-0.287190000	1.130867000	-0.190281000
C	2.488401000	-1.449892000	-0.345340000
C	1.792608000	-2.687688000	0.068027000
C	2.447284000	-3.967329000	-0.220497000
H	1.972614000	-4.847812000	0.210587000
C	3.594896000	-4.078709000	-0.924016000
C	4.233133000	-2.844422000	-1.491627000
H	5.309115000	-2.816940000	-1.237277000
C	3.600553000	-1.553835000	-1.102391000
C	3.994011000	1.028392000	0.406140000
C	4.877323000	1.722676000	-0.427880000
C	6.235258000	1.818149000	-0.118531000
H	6.878897000	2.382664000	-0.792690000
C	6.763859000	1.225852000	1.038173000
C	5.874568000	0.498496000	1.850525000
H	6.241532000	0.012402000	2.757113000
C	4.518944000	0.398109000	1.542890000
C	-1.748143000	1.195262000	-0.456214000
C	-2.602675000	1.976424000	0.342409000
C	-3.952056000	2.100355000	0.039828000
H	-4.574008000	2.724741000	0.684202000
C	-4.521147000	1.457407000	-1.076622000
C	-3.674317000	0.649440000	-1.848027000
H	-4.060873000	0.120659000	-2.718966000
C	-2.313747000	0.520772000	-1.545365000
H	-1.678491000	-0.086218000	-2.194386000
C	-0.332631000	-1.394256000	-0.135177000
C	0.205930000	-2.503564000	-0.907954000
C	-0.443227000	-3.789535000	-0.856008000
H	-0.084935000	-4.574213000	-1.525615000
C	-1.426957000	-4.045476000	0.066066000
H	-1.885125000	-5.032282000	0.155468000
C	-1.824614000	-2.995170000	0.916517000
C	-1.316432000	-1.686815000	0.810272000
C	-2.819993000	-3.091759000	2.037791000
H	-2.666717000	-4.019742000	2.609417000
C	-2.457612000	-1.842689000	2.927364000
C	-1.928312000	-0.827174000	1.883231000
H	-1.223700000	-0.096606000	2.307637000
H	-2.765720000	-0.252682000	1.462284000
H	0.658924000	-2.251793000	-1.869446000
H	1.355167000	-2.663426000	1.072310000
H	4.233109000	-2.903624000	-2.597752000
H	4.079713000	-0.652073000	-1.484385000
H	3.850747000	-0.154821000	2.207899000
H	4.496322000	2.215520000	-1.323967000
H	-2.202108000	2.491581000	1.215287000
C	4.316046000	-5.412124000	-1.126442000
C	-5.995899000	1.700814000	-1.420572000
C	3.442807000	-6.608648000	-0.717263000
H	2.499430000	-6.632508000	-1.285181000
H	3.984937000	-7.545001000	-0.919332000
H	3.200058000	-6.592557000	0.356006000

C	5.596910000	-5.418709000	-0.259377000
H	6.286312000	-4.607088000	-0.537247000
H	5.346824000	-5.309729000	0.807575000
H	6.131231000	-6.372733000	-0.393259000
C	4.705719000	-5.575651000	-2.612058000
H	5.181691000	-6.557099000	-2.762535000
H	3.818071000	-5.523014000	-3.263047000
H	5.421144000	-4.806981000	-2.939429000
C	-6.449874000	0.886474000	-2.641539000
H	-7.513293000	1.086854000	-2.844453000
H	-5.880714000	1.154393000	-3.545160000
H	-6.338544000	-0.196204000	-2.476540000
C	-6.883236000	1.321598000	-0.215700000
H	-6.646859000	1.927024000	0.672241000
H	-7.944666000	1.487912000	-0.460948000
H	-6.754257000	0.261805000	0.050866000
C	-6.180478000	3.201616000	-1.739777000
H	-7.231877000	3.409404000	-1.997217000
H	-5.910076000	3.834823000	-0.880741000
H	-5.551334000	3.500197000	-2.593705000
C	2.624532000	3.512753000	0.440772000
C	3.264311000	3.703550000	1.674067000
C	2.821362000	4.468738000	-0.559792000
C	4.109359000	4.790579000	1.877536000
C	3.676627000	5.555006000	-0.354028000
C	4.357113000	5.730379000	0.860193000
H	3.119705000	2.974169000	2.473712000
H	2.325864000	4.348046000	-1.525487000
H	4.603445000	4.893343000	2.845931000
H	3.819994000	6.260454000	-1.172330000
C	-0.223078000	3.649802000	-0.175476000
C	-0.305424000	4.540833000	0.903763000
C	-0.819035000	4.021851000	-1.385025000
C	-0.991142000	5.746801000	0.778163000
C	-1.497024000	5.236433000	-1.509970000
C	-1.610973000	6.122411000	-0.428414000
H	0.156749000	4.276751000	1.857197000
H	-0.765351000	3.344960000	-2.240560000
H	-1.051083000	6.405496000	1.647293000
H	-1.948122000	5.479309000	-2.471759000
C	-4.242210000	-3.125183000	1.498253000
C	-5.208061000	-3.902019000	2.155551000
C	-4.627030000	-2.388159000	0.366901000
C	-6.529842000	-3.935370000	1.703856000
H	-4.918443000	-4.486326000	3.032946000
C	-5.945983000	-2.430033000	-0.092858000
H	-3.899702000	-1.777029000	-0.170920000
C	-6.903479000	-3.199647000	0.574735000
H	-7.268360000	-4.544488000	2.231475000
H	-6.223528000	-1.859796000	-0.981256000
H	-7.934286000	-3.229839000	0.213209000
C	-2.391399000	7.441641000	-0.511449000
C	-3.581905000	7.374686000	0.470063000
H	-4.252109000	6.538928000	0.210881000
H	-3.244295000	7.230898000	1.507886000
H	-4.165283000	8.309176000	0.429246000
C	-1.467128000	8.615468000	-0.125205000

H	-1.076853000	8.510393000	0.898358000
H	-0.607818000	8.679034000	-0.812145000
H	-2.019491000	9.567829000	-0.177865000
C	-2.941424000	7.701523000	-1.922582000
H	-2.134609000	7.758010000	-2.670026000
H	-3.646637000	6.916263000	-2.236416000
H	-3.481475000	8.661073000	-1.937470000
C	5.367778000	6.860603000	1.097548000
C	6.756310000	6.232404000	1.353110000
H	6.748300000	5.572799000	2.234502000
H	7.079863000	5.633378000	0.486427000
H	7.507177000	7.020612000	1.526872000
C	4.943013000	7.689625000	2.327904000
H	3.951930000	8.144499000	2.169568000
H	4.893875000	7.074361000	3.239012000
H	5.668007000	8.499972000	2.507991000
C	5.475800000	7.804799000	-0.109611000
H	5.816191000	7.275135000	-1.012964000
H	4.511878000	8.286945000	-0.336354000
H	6.206326000	8.599956000	0.106635000
C	8.237749000	1.362582000	1.443952000
C	8.314965000	2.059575000	2.819790000
H	7.858437000	3.061629000	2.774321000
H	7.792836000	1.482904000	3.598327000
H	9.366071000	2.174097000	3.131351000
C	8.876585000	-0.039412000	1.537013000
H	9.936075000	0.042388000	1.829846000
H	8.369018000	-0.669965000	2.282822000
H	8.827859000	-0.555490000	0.564620000
C	9.040299000	2.198929000	0.435198000
H	10.087516000	2.272440000	0.767505000
H	9.037861000	1.743190000	-0.567308000
H	8.643161000	3.222574000	0.348939000
C	-1.321536000	-2.247618000	3.879721000
H	-1.660613000	-3.022958000	4.585090000
H	-0.455083000	-2.646642000	3.327178000
H	-0.978880000	-1.377679000	4.461853000
C	-3.636163000	-1.286557000	3.723952000
H	-4.450156000	-0.951767000	3.063875000
H	-4.044050000	-2.045750000	4.410218000
H	-3.310005000	-0.424368000	4.327601000

TS1(S-P)+: G = -2826.78227392 (i = -314 cm-1)

C	0.091465000	0.104150000	-0.403039000
C	1.483918000	0.307873000	-0.299377000
C	1.963793000	1.590080000	0.049693000
C	1.052937000	2.627767000	0.331849000
C	-0.336250000	2.438651000	0.146098000
C	-0.815650000	1.191248000	-0.296459000
C	2.417323000	-0.795790000	-0.649122000
C	2.220659000	-1.539363000	-1.821467000
C	3.088771000	-2.575154000	-2.179717000
H	2.891830000	-3.114527000	-3.105846000
C	4.192410000	-2.909116000	-1.383795000
C	4.385603000	-2.157970000	-0.208772000
C	3.525231000	-1.128277000	0.151907000

C	3.427893000	1.861348000	0.110250000
C	4.209534000	1.802904000	-1.052242000
C	5.577467000	2.059418000	-1.003781000
H	6.151211000	2.010522000	-1.931748000
C	6.226162000	2.374238000	0.204504000
C	5.434236000	2.431259000	1.361222000
H	5.883830000	2.670747000	2.324625000
C	4.059696000	2.185372000	1.315023000
C	-2.213147000	0.980200000	-0.755343000
C	-2.879845000	1.896930000	-1.487457000
C	-4.220482000	1.674931000	-2.096640000
H	-4.121684000	1.803534000	-3.192196000
C	-4.858547000	0.348781000	-1.798639000
C	-4.161782000	-0.582572000	-1.113708000
H	-4.609512000	-1.544895000	-0.869291000
C	-2.805664000	-0.367562000	-0.593914000
H	-2.678558000	-0.808992000	0.400483000
C	-0.488546000	-1.242052000	-0.571845000
C	-1.576714000	-1.411236000	-1.520482000
C	-2.178844000	-2.714468000	-1.679182000
H	-2.911865000	-2.849303000	-2.477364000
C	-1.894712000	-3.731836000	-0.805061000
H	-2.389768000	-4.702211000	-0.876775000
C	-0.956767000	-3.485342000	0.220478000
C	-0.252207000	-2.274789000	0.336552000
C	-0.645298000	-4.404567000	1.367406000
H	-0.460907000	-5.425061000	0.997144000
C	0.681841000	-3.796229000	1.958681000
C	0.575426000	-2.291023000	1.596410000
H	1.562274000	-1.830166000	1.489193000
H	0.040203000	-1.725827000	2.379608000
H	-1.525132000	-0.810416000	-2.431690000
H	1.390504000	-1.290800000	-2.485701000
H	5.226591000	-2.385658000	0.449036000
H	3.708481000	-0.581666000	1.076802000
H	3.471366000	2.241141000	2.233264000
H	3.737728000	1.552846000	-2.005256000
H	-2.425143000	2.869669000	-1.677849000
H	-4.897436000	2.499088000	-1.803150000
C	5.175727000	-4.030362000	-1.743254000
C	-6.307697000	0.138808000	-2.240252000
C	4.785156000	-4.750635000	-3.042896000
H	5.515120000	-5.547110000	-3.255170000
H	3.790792000	-5.218096000	-2.966869000
H	4.778124000	-4.064966000	-3.904504000
C	6.583864000	-3.422860000	-1.924059000
H	6.937996000	-2.934686000	-1.003404000
H	7.307786000	-4.210777000	-2.188823000
H	6.583666000	-2.671274000	-2.729678000
C	5.204955000	-5.069103000	-0.601558000
H	4.210875000	-5.520400000	-0.456543000
H	5.916718000	-5.875800000	-0.840913000
H	5.516011000	-4.618302000	0.352800000
C	-6.463442000	0.537299000	-3.724547000
H	-5.782634000	-0.046785000	-4.364737000
H	-7.495578000	0.339672000	-4.053545000
H	-6.261853000	1.606098000	-3.888728000

C	-7.225552000	1.031290000	-1.372910000
H	-8.275344000	0.891437000	-1.676248000
H	-7.136182000	0.765606000	-0.307816000
H	-6.985464000	2.099579000	-1.483323000
C	-6.754268000	-1.322694000	-2.078609000
H	-6.743686000	-1.641718000	-1.025343000
H	-7.785482000	-1.434712000	-2.447004000
H	-6.111448000	-2.007521000	-2.653807000
C	1.553506000	3.952831000	0.797962000
C	2.330385000	4.769059000	-0.036413000
C	1.234469000	4.427125000	2.074048000
C	2.755212000	6.023930000	0.391985000
C	1.668893000	5.683978000	2.502836000
C	2.432773000	6.515351000	1.670609000
H	2.595000000	4.419886000	-1.036486000
H	0.629814000	3.810060000	2.742108000
H	3.348668000	6.634980000	-0.291483000
H	1.395178000	6.010705000	3.505857000
C	-1.268398000	3.557805000	0.463163000
C	-1.186990000	4.792922000	-0.197575000
C	-2.225023000	3.414896000	1.473370000
C	-2.024137000	5.847091000	0.156450000
C	-3.058851000	4.478315000	1.831138000
C	-2.973338000	5.721925000	1.188021000
H	-0.453699000	4.931311000	-0.994032000
H	-2.309573000	2.462047000	2.001868000
H	-1.925843000	6.792891000	-0.380617000
H	-3.777515000	4.322553000	2.635555000
C	-1.818161000	-4.484800000	2.333141000
C	-2.152597000	-5.716249000	2.914492000
C	-2.563648000	-3.349296000	2.689343000
C	-3.198914000	-5.812652000	3.836433000
H	-1.583065000	-6.608829000	2.642258000
C	-3.610681000	-3.442359000	3.609616000
H	-2.329294000	-2.378993000	2.247214000
C	-3.931901000	-4.674924000	4.187669000
H	-3.443632000	-6.781478000	4.279459000
H	-4.179406000	-2.547278000	3.874184000
H	-4.752304000	-4.748642000	4.905937000
C	-3.849071000	6.923109000	1.569592000
C	-4.810738000	6.596050000	2.722440000
H	-5.503483000	5.782625000	2.455576000
H	-4.269286000	6.301061000	3.634863000
H	-5.414487000	7.484867000	2.963359000
C	-2.937552000	8.089787000	2.008774000
H	-3.546016000	8.963517000	2.293838000
H	-2.323445000	7.800422000	2.876773000
H	-2.257542000	8.400845000	1.201270000
C	-4.685587000	7.353866000	0.345799000
H	-4.048194000	7.648309000	-0.501855000
H	-5.339506000	6.532233000	0.011859000
H	-5.322157000	8.216001000	0.603618000
C	7.736918000	2.645660000	0.213464000
C	8.270438000	2.911515000	1.629589000
H	7.805819000	3.801321000	2.082471000
H	8.094515000	2.053977000	2.297834000
H	9.356635000	3.087500000	1.587032000

C	8.029786000	3.884873000	-0.659841000
H	9.111259000	4.098080000	-0.670359000
H	7.704867000	3.734230000	-1.700812000
H	7.508240000	4.771909000	-0.265440000
C	8.479489000	1.421059000	-0.363104000
H	9.567270000	1.598590000	-0.358764000
H	8.275958000	0.521486000	0.239837000
H	8.177555000	1.211209000	-1.400218000
C	2.907760000	7.912160000	2.094000000
C	2.472025000	8.264986000	3.524566000
H	1.375515000	8.270252000	3.627018000
H	2.882866000	7.558594000	4.262815000
H	2.837322000	9.270988000	3.783509000
C	4.448859000	7.968302000	2.023924000
H	4.897855000	7.227192000	2.704666000
H	4.817622000	7.762380000	1.007606000
H	4.808727000	8.967923000	2.317659000
C	2.307836000	8.959693000	1.131000000
H	2.625604000	8.786496000	0.091505000
H	1.206579000	8.929156000	1.159637000
H	2.631600000	9.973268000	1.419007000
C	0.856159000	-4.023188000	3.458944000
H	0.042449000	-3.562510000	4.038092000
H	0.875255000	-5.098981000	3.695844000
H	1.808252000	-3.583916000	3.797345000
C	1.864429000	-4.411438000	1.193571000
H	2.813752000	-3.964491000	1.522597000
H	1.914585000	-5.498914000	1.362712000
H	1.780160000	-4.236729000	0.109373000

P1(S-M)+: G = -2826.78606801

C	0.382362000	-0.148512000	-0.072266000
C	1.810084000	-0.186718000	-0.079934000
C	2.521951000	1.002880000	0.157157000
C	1.829270000	2.235063000	0.231059000
C	0.438318000	2.291034000	0.019017000
C	-0.300957000	1.094492000	-0.103891000
C	2.484156000	-1.472125000	-0.373487000
C	1.762660000	-2.723530000	0.040689000
C	2.473703000	-3.996976000	-0.285810000
H	2.029506000	-4.896405000	0.140717000
C	3.614230000	-4.079343000	-0.991116000
C	4.234386000	-2.830938000	-1.563497000
H	5.311232000	-2.788954000	-1.316325000
C	3.586179000	-1.553754000	-1.138602000
C	3.998035000	1.013643000	0.375835000
C	4.854235000	1.720795000	-0.474839000
C	6.215641000	1.840134000	-0.189232000
H	6.837702000	2.414473000	-0.875232000
C	6.774234000	1.259752000	0.959281000
C	5.911808000	0.518440000	1.787989000
H	6.302300000	0.039975000	2.688832000
C	4.553086000	0.394544000	1.504195000
C	-1.752335000	1.153135000	-0.408338000
C	-2.632418000	1.967315000	0.328489000
C	-3.970356000	2.076900000	-0.023837000

H	-4.614640000	2.722817000	0.575594000
C	-4.502096000	1.390492000	-1.133590000
C	-3.629929000	0.556113000	-1.846268000
H	-3.986553000	-0.003117000	-2.710824000
C	-2.281203000	0.439087000	-1.492205000
H	-1.623835000	-0.188667000	-2.097932000
C	-0.323423000	-1.426852000	0.025487000
C	0.286628000	-2.601418000	-0.652728000
C	-0.421120000	-3.887779000	-0.563974000
H	-0.030813000	-4.714451000	-1.161158000
C	-1.473324000	-4.069858000	0.278688000
H	-1.971302000	-5.035253000	0.384878000
C	-1.884749000	-2.965316000	1.066230000
C	-1.368106000	-1.664463000	0.920620000
C	-2.916453000	-3.015218000	2.151176000
H	-2.776345000	-3.919500000	2.763407000
C	-2.598296000	-1.725771000	2.995257000
C	-2.029329000	-0.759607000	1.926076000
H	-1.345426000	-0.005374000	2.342163000
H	-2.851848000	-0.210909000	1.447179000
H	0.516829000	-2.377401000	-1.706387000
H	1.544839000	-2.709944000	1.122322000
H	4.219157000	-2.878516000	-2.668850000
H	4.051580000	-0.639694000	-1.508500000
H	3.905563000	-0.167793000	2.181632000
H	4.449592000	2.206004000	-1.364829000
H	-2.262079000	2.512080000	1.196283000
C	4.357080000	-5.400840000	-1.216544000
C	-5.966492000	1.612768000	-1.531414000
C	3.513342000	-6.617762000	-0.805779000
H	2.564183000	-6.656605000	-1.363223000
H	4.071169000	-7.542593000	-1.019297000
H	3.281306000	-6.613027000	0.270112000
C	5.651219000	-5.396500000	-0.370827000
H	6.320482000	-4.567153000	-0.645914000
H	5.415626000	-5.307308000	0.701445000
H	6.204218000	-6.337026000	-0.525292000
C	4.725871000	-5.547094000	-2.708800000
H	5.215486000	-6.519075000	-2.878045000
H	3.827038000	-5.502518000	-3.345233000
H	5.422629000	-4.763648000	-3.041658000
C	-6.374351000	0.763315000	-2.744470000
H	-7.430777000	0.952867000	-2.989722000
H	-5.774897000	1.009395000	-3.634596000
H	-6.265028000	-0.313786000	-2.545984000
C	-6.887218000	1.255892000	-0.344739000
H	-6.682495000	1.885450000	0.534275000
H	-7.941805000	1.406856000	-0.626523000
H	-6.757997000	0.204143000	-0.048217000
C	-6.154328000	3.102854000	-1.894922000
H	-7.199565000	3.293362000	-2.188076000
H	-5.915458000	3.760348000	-1.045117000
H	-5.503321000	3.385331000	-2.737830000
C	2.612459000	3.477219000	0.486677000
C	3.272736000	3.652747000	1.711418000
C	2.784947000	4.449298000	-0.502863000
C	4.112774000	4.743133000	1.916936000

C	3.636268000	5.538050000	-0.295136000
C	4.335778000	5.700253000	0.910025000
H	3.147909000	2.909860000	2.501814000
H	2.273853000	4.340178000	-1.461561000
H	4.622406000	4.835063000	2.878303000
H	3.760584000	6.256771000	-1.104922000
C	-0.230708000	3.617701000	-0.104136000
C	-0.313189000	4.500794000	0.981346000
C	-0.799094000	4.010165000	-1.320100000
C	-0.966831000	5.724081000	0.852942000
C	-1.445819000	5.241688000	-1.447146000
C	-1.553027000	6.124584000	-0.362340000
H	0.128220000	4.219649000	1.939838000
H	-0.745153000	3.338677000	-2.179983000
H	-1.023688000	6.379169000	1.724974000
H	-1.873669000	5.502688000	-2.414815000
C	-4.313805000	-3.094308000	1.552073000
C	-5.292138000	-3.866532000	2.196128000
C	-4.660339000	-2.406017000	0.378609000
C	-6.592011000	-3.940430000	1.689868000
H	-5.030260000	-4.412513000	3.106146000
C	-5.956919000	-2.490752000	-0.135633000
H	-3.922531000	-1.798705000	-0.148972000
C	-6.928313000	-3.253926000	0.518587000
H	-7.342329000	-4.543378000	2.207677000
H	-6.205318000	-1.959816000	-1.056295000
H	-7.941989000	-3.316120000	0.115286000
C	-2.286144000	7.470151000	-0.452584000
C	-3.486589000	7.450430000	0.518721000
H	-4.186320000	6.640773000	0.255386000
H	-3.164249000	7.296668000	1.559909000
H	-4.032763000	8.406713000	0.470347000
C	-1.321964000	8.609250000	-0.059237000
H	-0.946902000	8.491213000	0.968559000
H	-0.453566000	8.638895000	-0.737007000
H	-1.837344000	9.581709000	-0.119291000
C	-2.813851000	7.748613000	-1.868682000
H	-1.998644000	7.779540000	-2.608434000
H	-3.540985000	6.986665000	-2.189960000
H	-3.322340000	8.725152000	-1.887442000
C	5.339959000	6.835904000	1.148303000
C	6.737572000	6.216905000	1.374940000
H	6.748591000	5.545163000	2.247064000
H	7.054101000	5.633007000	0.495452000
H	7.483592000	7.009637000	1.548812000
C	4.925660000	7.643685000	2.396280000
H	3.928418000	8.091782000	2.258674000
H	4.895221000	7.015187000	3.299130000
H	5.646087000	8.457855000	2.577325000
C	5.421627000	7.797728000	-0.046916000
H	5.752006000	7.283558000	-0.962858000
H	4.450555000	8.275297000	-0.251807000
H	6.149139000	8.595660000	0.169198000
C	8.252085000	1.423695000	1.340090000
C	8.339383000	2.128342000	2.711425000
H	7.865015000	3.122164000	2.668844000
H	7.839746000	1.546442000	3.500778000

H	9.393220000	2.262434000	3.005461000
C	8.916397000	0.033392000	1.428780000
H	9.978783000	0.134284000	1.704776000
H	8.431045000	-0.602390000	2.184775000
H	8.861358000	-0.487573000	0.459351000
C	9.023513000	2.269214000	0.314872000
H	10.074487000	2.362811000	0.629889000
H	9.012982000	1.808987000	-0.685534000
H	8.607154000	3.285341000	0.230893000
C	-1.504580000	-2.078496000	4.015048000
H	-1.872003000	-2.821519000	4.740667000
H	-0.612314000	-2.497929000	3.521724000
H	-1.192281000	-1.179822000	4.569810000
C	-3.814210000	-1.137700000	3.708625000
H	-4.600746000	-0.848199000	2.996155000
H	-4.245562000	-1.861473000	4.418332000
H	-3.519904000	-0.240177000	4.275806000

P1(S-P)+: G = -2826.78313721

C	0.079772000	0.070991000	-0.374954000
C	1.477171000	0.272893000	-0.264771000
C	1.956693000	1.554744000	0.077868000
C	1.041603000	2.591346000	0.351357000
C	-0.344305000	2.413398000	0.127059000
C	-0.825150000	1.169055000	-0.322660000
C	2.407960000	-0.823411000	-0.636858000
C	2.183277000	-1.568027000	-1.804490000
C	3.054288000	-2.589671000	-2.194564000
H	2.835246000	-3.128385000	-3.116042000
C	4.189443000	-2.907583000	-1.437651000
C	4.409333000	-2.157744000	-0.265949000
C	3.546975000	-1.142465000	0.126546000
C	3.419810000	1.831708000	0.131754000
C	4.192343000	1.778665000	-1.037156000
C	5.563562000	2.016769000	-0.995039000
H	6.130984000	1.967793000	-1.926836000
C	6.224215000	2.308095000	0.212671000
C	5.439385000	2.370575000	1.373952000
H	5.897958000	2.596446000	2.336412000
C	4.061190000	2.142732000	1.334620000
C	-2.213061000	0.958411000	-0.800308000
C	-2.882146000	1.888850000	-1.503995000
C	-4.223474000	1.685336000	-2.128530000
H	-4.124297000	1.838455000	-3.219878000
C	-4.857477000	0.346470000	-1.855194000
C	-4.161622000	-0.602383000	-1.206521000
H	-4.619287000	-1.567616000	-0.990858000
C	-2.781104000	-0.426692000	-0.666310000
H	-2.769600000	-0.760096000	0.384492000
C	-0.502436000	-1.267095000	-0.503649000
C	-1.674379000	-1.419314000	-1.394820000
C	-2.241593000	-2.765970000	-1.549176000
H	-3.015100000	-2.902518000	-2.307791000
C	-1.877108000	-3.789742000	-0.729893000
H	-2.337317000	-4.777106000	-0.798850000
C	-0.905809000	-3.525487000	0.270255000

C	-0.204115000	-2.312061000	0.370681000
C	-0.565745000	-4.441235000	1.406224000
H	-0.405889000	-5.465643000	1.035028000
C	0.777506000	-3.841055000	1.961564000
C	0.666024000	-2.335956000	1.603060000
H	1.651653000	-1.883174000	1.465290000
H	0.159206000	-1.766509000	2.401701000
H	-1.517766000	-0.945971000	-2.374805000
H	1.331687000	-1.326526000	-2.443820000
H	5.274845000	-2.374714000	0.362913000
H	3.752674000	-0.597851000	1.047551000
H	3.479216000	2.196757000	2.257088000
H	3.711669000	1.540806000	-1.988858000
H	-2.428761000	2.866955000	-1.667386000
H	-4.898956000	2.500658000	-1.809646000
C	5.179804000	-4.009078000	-1.834566000
C	-6.309655000	0.149371000	-2.302503000
C	4.761092000	-4.728504000	-3.125811000
H	5.499362000	-5.508616000	-3.367814000
H	3.779436000	-5.216557000	-3.020032000
H	4.711641000	-4.037462000	-3.981695000
C	6.570826000	-3.375552000	-2.055067000
H	6.943109000	-2.882032000	-1.144432000
H	7.300282000	-4.150344000	-2.342117000
H	6.533602000	-2.623168000	-2.859082000
C	5.262199000	-5.053513000	-0.700458000
H	4.281161000	-5.523688000	-0.528851000
H	5.981019000	-5.845269000	-0.966754000
H	5.593684000	-4.602655000	0.246959000
C	-6.462772000	0.568229000	-3.781033000
H	-5.783936000	-0.010765000	-4.428199000
H	-7.495395000	0.381640000	-4.115762000
H	-6.254067000	1.637773000	-3.931489000
C	-7.227348000	1.031504000	-1.425475000
H	-8.277254000	0.906063000	-1.735540000
H	-7.145339000	0.745987000	-0.364764000
H	-6.979570000	2.100124000	-1.513830000
C	-6.766036000	-1.311362000	-2.163694000
H	-6.752996000	-1.648057000	-1.115840000
H	-7.799603000	-1.411291000	-2.529616000
H	-6.130108000	-1.991274000	-2.752353000
C	1.523450000	3.914309000	0.841518000
C	2.283768000	4.763240000	0.025411000
C	1.163220000	4.368113000	2.114375000
C	2.631223000	6.040205000	0.459507000
C	1.521093000	5.646293000	2.548752000
C	2.246363000	6.519897000	1.725209000
H	2.583512000	4.428497000	-0.969723000
H	0.573904000	3.723062000	2.769359000
H	3.203706000	6.681994000	-0.213600000
H	1.208862000	5.959348000	3.544749000
C	-1.258035000	3.555367000	0.419778000
C	-1.143137000	4.775067000	-0.263897000
C	-2.197167000	3.465419000	1.451368000
C	-1.917741000	5.872183000	0.100447000
C	-2.970828000	4.571113000	1.817349000
C	-2.838187000	5.804575000	1.162783000

H	-0.425094000	4.870110000	-1.080380000
H	-2.308732000	2.524436000	1.995920000
H	-1.787270000	6.807381000	-0.448121000
H	-3.674803000	4.457946000	2.641665000
C	-1.722957000	-4.499414000	2.394919000
C	-2.052752000	-5.721653000	2.998093000
C	-2.454616000	-3.353749000	2.747932000
C	-3.080581000	-5.798471000	3.941855000
H	-1.493667000	-6.621013000	2.727006000
C	-3.484101000	-3.428900000	3.689645000
H	-2.226338000	-2.390344000	2.287183000
C	-3.799921000	-4.651271000	4.291230000
H	-3.321615000	-6.759323000	4.403718000
H	-4.043277000	-2.527328000	3.952247000
H	-4.606217000	-4.710222000	5.026572000
C	-3.624662000	7.057809000	1.569869000
C	-4.453720000	7.557404000	0.367601000
H	-3.814968000	7.814388000	-0.491017000
H	-5.170483000	6.786841000	0.041020000
H	-5.022958000	8.459590000	0.645055000
C	-4.582893000	6.786440000	2.739852000
H	-5.329864000	6.018799000	2.483637000
H	-4.043748000	6.454476000	3.640840000
H	-5.124940000	7.710326000	2.995168000
C	-2.625267000	8.154401000	1.999913000
H	-3.165241000	9.065115000	2.306491000
H	-2.013560000	7.814013000	2.851052000
H	-1.941811000	8.426438000	1.180959000
C	2.596295000	7.955939000	2.138372000
C	2.130812000	8.278301000	3.566822000
H	1.037857000	8.190183000	3.669232000
H	2.600409000	7.613755000	4.308984000
H	2.407343000	9.313620000	3.820243000
C	4.124507000	8.157839000	2.064450000
H	4.643244000	7.467518000	2.749014000
H	4.510407000	7.983539000	1.048703000
H	4.387313000	9.188878000	2.352114000
C	1.897054000	8.933096000	1.167625000
H	2.225607000	8.777596000	0.128628000
H	0.803486000	8.800145000	1.202311000
H	2.126039000	9.975658000	1.443087000
C	7.741321000	2.541624000	0.216387000
C	8.286518000	2.792036000	1.630820000
H	7.844201000	3.691447000	2.087035000
H	8.093582000	1.937517000	2.298212000
H	9.376312000	2.942793000	1.584170000
C	8.447891000	1.297185000	-0.363757000
H	9.540356000	1.443315000	-0.360690000
H	8.219288000	0.402210000	0.237155000
H	8.138264000	1.098646000	-1.400842000
C	8.065178000	3.772532000	-0.657342000
H	9.152091000	3.955727000	-0.671087000
H	7.733404000	3.631763000	-1.697484000
H	7.569784000	4.673453000	-0.260925000
C	0.990560000	-4.069562000	3.456419000
H	0.192805000	-3.608297000	4.056937000
H	1.014930000	-5.145590000	3.691530000

H	1.951729000	-3.631391000	3.769318000
C	1.935260000	-4.460553000	1.162837000
H	2.895727000	-4.021127000	1.468636000
H	1.982494000	-5.549083000	1.325662000
H	1.822855000	-4.280290000	0.081946000

(S,M)-4: G = -2820.68041741

C	0.492991000	-0.173946000	0.112757000
C	1.911216000	-0.190546000	0.070384000
C	2.644901000	1.024350000	0.142704000
C	1.952620000	2.249544000	0.276062000
C	0.542516000	2.271698000	0.112196000
C	-0.166510000	1.066257000	-0.119095000
C	2.608427000	-1.437128000	-0.122212000
C	1.880024000	-2.653068000	-0.215116000
C	2.551428000	-3.823043000	-0.605922000
H	1.980661000	-4.741027000	-0.714905000
C	3.925572000	-3.841013000	-0.858621000
C	4.639441000	-2.648539000	-0.682045000
C	4.019399000	-1.449094000	-0.308913000
C	4.082984000	1.005002000	0.123510000
C	4.780735000	-0.211290000	-0.109200000
C	6.184259000	-0.199631000	-0.129950000
H	6.712001000	-1.134407000	-0.293967000
C	6.922820000	0.969835000	0.066991000
C	6.217584000	2.157689000	0.294888000
H	6.783154000	3.074757000	0.446098000
C	4.817826000	2.207766000	0.332971000
C	-1.513186000	1.169101000	-0.670792000
C	-2.093472000	0.124746000	-1.420488000
C	-3.383963000	0.216779000	-1.916175000
H	-3.782473000	-0.621758000	-2.486138000
C	-4.172774000	1.364358000	-1.687185000
C	-3.566721000	2.427913000	-1.020391000
H	-4.130120000	3.348562000	-0.888769000
C	-2.244227000	2.375483000	-0.536158000
C	-0.192077000	-1.424358000	0.450367000
C	0.477047000	-2.661590000	0.208983000
C	-0.175359000	-3.877278000	0.511578000
H	0.328723000	-4.823478000	0.316446000
C	-1.416561000	-3.899840000	1.130455000
H	-1.892451000	-4.850985000	1.382440000
C	-2.006764000	-2.687505000	1.498885000
C	-1.422058000	-1.467147000	1.159355000
C	-3.264904000	-2.500562000	2.315470000
H	-3.284979000	-3.203356000	3.163646000
C	-3.078379000	-1.034107000	2.870557000
C	-2.240120000	-0.349612000	1.761838000
H	-1.625641000	0.478736000	2.146932000
H	-2.915032000	0.088510000	1.012866000
H	5.712753000	-2.654011000	-0.861658000
H	-1.515555000	-0.778098000	-1.614599000
C	4.669887000	-5.108032000	-1.306338000
C	-5.646131000	1.439946000	-2.112104000
C	3.734201000	-6.320889000	-1.425110000

H	2.939245000	-6.153500000	-2.168594000
H	4.311964000	-7.201382000	-1.746601000
H	3.258427000	-6.565267000	-0.462545000
C	5.770630000	-5.441911000	-0.276501000
H	6.501762000	-4.624923000	-0.180421000
H	5.332820000	-5.625809000	0.717759000
H	6.317500000	-6.348182000	-0.584021000
C	5.315665000	-4.853097000	-2.685240000
H	5.852750000	-5.753053000	-3.026767000
H	4.548689000	-4.604738000	-3.436311000
H	6.037656000	-4.023165000	-2.648939000
C	-6.020777000	2.857162000	-2.588029000
H	-7.066268000	2.868104000	-2.934469000
H	-5.937184000	3.603517000	-1.784305000
H	-5.379070000	3.178968000	-3.424043000
C	-5.961296000	0.456619000	-3.253193000
H	-5.826610000	-0.590725000	-2.948045000
H	-7.011084000	0.574295000	-3.563931000
H	-5.321956000	0.642983000	-4.130941000
C	-6.505709000	1.074846000	-0.880128000
H	-7.578042000	1.101058000	-1.136214000
H	-6.262894000	0.064609000	-0.517281000
H	-6.330142000	1.784930000	-0.056522000
C	2.674462000	3.468596000	0.522993000
C	4.094981000	3.459478000	0.581822000
C	1.969866000	4.692000000	0.717186000
C	4.771928000	4.656986000	0.868079000
C	2.700261000	5.854020000	0.992607000
C	4.098613000	5.860949000	1.082118000
H	5.855974000	4.643963000	0.926593000
H	2.167692000	6.791821000	1.136516000
C	-0.175546000	3.517564000	0.228483000
C	0.506497000	4.705176000	0.605712000
C	-1.585969000	3.553743000	0.032974000
C	-0.242050000	5.867492000	0.859154000
C	-2.280908000	4.735825000	0.310322000
C	-1.632708000	5.902876000	0.736148000
H	0.279337000	6.766951000	1.172572000
H	-3.362761000	4.752672000	0.190181000
C	-4.536536000	-2.732420000	1.518586000
C	-5.694347000	-3.184533000	2.171599000
C	-4.603780000	-2.488972000	0.139064000
C	-6.888656000	-3.375939000	1.472047000
H	-5.655828000	-3.389148000	3.245231000
C	-5.794702000	-2.684348000	-0.566261000
H	-3.715232000	-2.144386000	-0.390495000
C	-6.943783000	-3.125621000	0.096662000
H	-7.777950000	-3.728700000	2.001308000
H	-5.822919000	-2.492009000	-1.641354000
H	-7.874518000	-3.279383000	-0.455416000
C	-2.459866000	7.162240000	1.034255000
C	-1.580477000	8.346740000	1.463786000
H	-1.015311000	8.125274000	2.382578000
H	-0.862127000	8.626647000	0.677547000
H	-2.215682000	9.223346000	1.664352000
C	-3.456065000	6.852957000	2.172355000
H	-4.144418000	6.038653000	1.899225000

H	-2.922525000	6.554762000	3.089128000
H	-4.062164000	7.744698000	2.401281000
C	-3.239667000	7.569607000	-0.234685000
H	-2.548562000	7.787021000	-1.064777000
H	-3.926528000	6.774943000	-0.563022000
H	-3.838699000	8.473980000	-0.039502000
C	4.830604000	7.174319000	1.396490000
C	6.352205000	6.982270000	1.492551000
H	6.623141000	6.266244000	2.284117000
H	6.780843000	6.624349000	0.543517000
H	6.829969000	7.944666000	1.732885000
C	4.324760000	7.728742000	2.745622000
H	3.244109000	7.935845000	2.722561000
H	4.517730000	7.012559000	3.560442000
H	4.842505000	8.671378000	2.986947000
C	4.536741000	8.197287000	0.277958000
H	4.885578000	7.821335000	-0.697212000
H	3.460260000	8.411822000	0.195078000
H	5.055067000	9.147604000	0.485937000
C	8.458759000	0.993123000	0.043626000
C	8.932215000	1.931312000	-1.087460000
H	10.033562000	1.960669000	-1.121902000
H	8.566852000	1.579718000	-2.065670000
H	8.572604000	2.961165000	-0.940362000
C	8.979949000	1.517499000	1.398937000
H	8.647459000	0.867184000	2.223830000
H	10.081935000	1.539045000	1.400231000
H	8.623101000	2.538058000	1.604694000
C	9.057305000	-0.401068000	-0.200457000
H	10.156212000	-0.333534000	-0.206883000
H	8.769749000	-1.113158000	0.588755000
H	8.742004000	-0.816599000	-1.170231000
C	-4.383627000	-0.282755000	3.131779000
H	-4.983574000	-0.180246000	2.215055000
H	-5.000044000	-0.798837000	3.885236000
H	-4.164901000	0.729672000	3.509281000
C	-2.258214000	-1.114756000	4.167611000
H	-2.818810000	-1.647059000	4.953137000
H	-1.308299000	-1.648451000	4.004977000
H	-2.021444000	-0.104734000	4.539413000

Stereoselectivity in Scholl reaction.

In a kinetically controlled reaction like this graphitization process, stereoselectivity and product distribution can be explained solely by the relative rates at which each product is formed.[15,16] Consequently, the macroscopic rate constant for the formation of each product (diastereoisomers A and B, for example) is governed by the activation free energy ($\Delta G^{\ddagger}$) of its corresponding transition state, as illustrated in the following equations:

[15] Q. Peng, F. Duarte, R. Paton, *Chem. Soc. Rev.*, 2016, **45**, 6093.
[16] P. Nakliang, S. Yoon, S. Choi, *Org. Chem. Front.*, 2021, **8**, 5165.

$$k_A = \frac{k_B T}{h} e^{-\Delta G_A^{\neq} / RT} \; ; \; k_B = \frac{k_B T}{h} e^{-\Delta G_B^{\neq} / RT} \; ; \; \frac{[A]}{[B]} = \frac{k_{[A]}}{k[B]} = exp^{-\Delta\Delta G^{\neq} / RT}$$

Here, $\Delta G_A^{\ddagger}$ and $\Delta G_B^{\ddagger}$ represent the activation free energies for formation of products A and B, respectively; $\Delta\Delta G^{\ddagger} = \Delta G_A^{\ddagger} - \Delta G_B^{\ddagger}$; k_B is the Boltzmann constant, *h* is Planck's constant, T is the temperature, and R is the gas constant.

Following this model, a computed $\Delta\Delta G^{\ddagger}$ of 2.1 kcal/mol at 195 K corresponds to a diastereomeric ratio greater than 99:1. This large kinetic preference is fully consistent with the exclusive formation of the (*S,M*)-**4** nanographene observed experimentally.
